# Intelligent Stain‐Free Histology on Structural Colorimetric Nanocavities

**DOI:** 10.1002/advs.202514340

**Published:** 2026-02-12

**Authors:** Qizhe Chen, Yifei Ren, Lijie Hu, Yanyan Li, Wenyue Liang, Jin Wang, Han Gao, Xinhai Wang, Jiajun Li, Qiutao He, Yingfeng Zhu, Haifeng Hu, Qiwen Zhan, Imed Gallouzi, Jasmeen Merzaban, Di Wang, Zunguo Du, Xiaodong Gu, Qiaoqiang Gan

**Affiliations:** ^1^ Material Science and Engineering Physical Science and Engineering Division King Abdullah University of Science and Technology Thuwal Saudi Arabia; ^2^ Department of Pathology Huashan Hospital Fudan University Shanghai China; ^3^ Computer Science Computer Electrical and Mathematical Sciences and Engineering Division King Abdullah University of Science and Technology Thuwal Saudi Arabia; ^4^ Bioscience Program Biological and Environmental Science and Engineering Division King Abdullah University of Science and Technology Thuwal Saudi Arabia; ^5^ School of Optical‐Electrical and Computer Engineering University of Shanghai for Science and Technology Shanghai China; ^6^ Department of General Surgery Huashan Hospital Fudan University Shanghai China; ^7^ KAUST Center of Excellence for Smart Health (KCSH) King Abdullah University of Science and Technology Thuwal Saudi Arabia; ^8^ Environmental Science and Engineering Biological and Environmental Science and Engineering Division King Abdullah University of Science and Technology Thuwal Saudi Arabia

**Keywords:** AI‐assisted diagnosis, on‐chip cancer diagnosis, nanocavities‐on‐silicon (NOS), stain‐free histology, structural color contrast

## Abstract

Optical microscopic imaging and analysis are indispensable in modern pathology for providing conclusive evidence in disease diagnosis. However, the current gold standard histological staining techniques, hematoxylin and eosin (H&E), can be inconsistent, costly, time‐consuming, and laborious. Although nanophotonic chips offer a promising stain‐free approach to revealing the optical features of healthy and cancerous tissue, their clinical adoption is hindered by the significant costs associated with top‐down lithography. This study introduces planar nanocavities‐on‐silicon (NOS) for nonstained histological imaging, eliminating the need for traditional staining and lateral nanopatterns. By depositing an approximately 270‐nm‐thick silicon nitride layer onto a standard silicon wafer, NOS slides produce vibrant structural colors via optical interference that is sensitive to local refractive index and thickness of tissue sections. This method simplifies the visualization of morphological features using conventional microscopes and reduces raw material and processing costs per test. Comparative imaging analyses between NOS and H&E‐stained slides show good agreement in key histological structures. Moreover, a deep‐learning model trained on over 2700 NOS images achieves >95% accuracy in distinguishing cancerous from healthy colorectal tissues. These results suggest NOS as a faster, inexpensive, chemical‐free, stain‐free platform that enables future AI‐assisted screening of colorectal cancer for improved pathological diagnostics.

## Graphical abstract

Chen and Gan et al. introduce a label‐free imaging platform using inexpensive Nanocavity‐on‐Silicon (NOS) slides. These slides transform subtle tissue variations into vivid structural colors, enabling high‐contrast histological imaging under a regular optical microscope. This stain‐free approach reveals morphological details comparable to traditional H&E staining and facilitates rapid AI‐powered diagnosis.

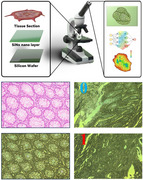

## 1 Main

Pathologists have traditionally relied on meticulously examining biological tissue stained with hematoxylin and eosin (H&E) to assess pathological features via morphological characteristics and color distributions [1]. While foundational, the efficacy of this method is significantly influenced by the consistency of the staining process and the expertise of the pathologist. The staining procedure is time‐consuming and susceptible to inconsistencies due to fluctuating staining solution concentrations and dye quality variability. Even with commercial auto‐sample‐preparation systems (e.g., the Leica ST5010 Autostainer), the quality of the resulting samples still depends on the uniformity and expiration date of the biochemical reagents [2]. Moreover, fresh and inexperienced pathologists face a steep learning curve in accurately identifying critical features, underscoring the substantial reliance of diagnostic criteria on individual expertise [3]. This subjective approach poses significant difficulties in the evolving landscape of digital and personalized medical diagnostics, especially in a global context where the cost of histological diagnosis dramatically varies due to differences in labor costs [4, 5]. Therefore, a shift toward accurate, consistent, standardized, and digital diagnostic criteria could transform diagnostic accuracy and efficiency globally [6], potentially making high‐quality healthcare services more accessible, particularly in regions with high labor costs. Such advancements could expedite the diagnostic process, leading to faster and more effective patient care while substantially reducing the financial burden of traditional diagnostic methods. For instance, stain‐free histological analysis has emerged as a pivotal innovation to reduce the costs and labor associated with pathological diagnostics [7]. Over the past decade, several methods have been developed, primarily in research settings. For example, mid‐infrared spectrochemical imaging employs optical absorption contrasts at various vibrational wavelengths [8, 9]. However, capturing tissue images using this method requires sophisticated quantum‐cascade‐laser‐based mid‐infrared microscopy. Virtual staining has also gained popularity in stain‐free histology to generate histological stains digitally [10, 11]. Nevertheless, its practical application in clinical settings is constrained by the challenge of obtaining numerous tissue images for training and validation because these images depend on advanced and expensive microscopy technology [12, 13, 14, 15, 16].

Recently, a promising approach employing plasmonic nanohole array slides was introduced for colorimetric histology on unlabeled specimens [17]. This method demonstrates that paraffin‐embedded tissue sections as thin as 1 to 5 µm mounted on nanohole array slides can distinctly differentiate cancerous areas from nonstained tissue using standard clinical optical bright‐field microscopes, which are typically inexpensive and widely available. However, the complex and costly fabrication of nanopatterns on metallic films is a significant barrier to widespread production and clinical implementation [18, 19, 20, 21, 22]. Therefore, these significant technical and cost barriers prevent the adoption of nanophotonic chips in practical clinical settings. Innovative techniques must align with the existing clinic settings and potentially surpass the cost‐efficiency of traditional H&E staining methods to gain traction in pathology diagnostics. This criterion is crucial for transitioning the stain‐free histological analysis from the research laboratory to practical clinical settings, effectively integrating technological advancements with the demands of medical practice.

This work presents an interdisciplinary approach combining advances in nanotechnology, optics, and artificial intelligence (AI) to overcome these challenges. We introduce an optical interferometric technique using planar nanocavities‐on‐silicon (NOS) for stain‐free histological imaging. Embedding an approximately 270‐nm‐thick dielectric nanocavity of silicon nitride (Si_3_N_4_) onto a standard silicon wafer produces NOS slides that generate optical interference structural colors, eliminating the need for traditional H&E stains. When tissue sections are mounted on NOS slides, slight adjustments in optical interference conditions occur due to minor variations in thickness or the refractive index (RI) of the cellular contents. Consequently, morphological features and color contrasts become readily observable under conventional microscopes. Compared with standard transmitted‐light brightfield platforms used for routine primary reads and whole‐slide imaging, NOS operates in reflection mode and can be implemented with simple, low‐cost microscope configuration updates, such as straightforward epi‐illumination add‐ons, making it well‐suited as an adjunct imaging pathway under current clinical hardware. This work demonstrates that NOS slides can reveal biological information comparable to H&E staining, with similar morphologies and color contrasts via serial sectioning and comparative image analyses of specimens on NOS and H&E‐stained slides. The structural color images produced on NOS slides enable experienced pathologists to distinguish between healthy and cancerous tissues. A critical advantage of this method is the cost‐effective production process of NOS slides, significantly reducing the raw material and processing costs per test (through a sample handling procedure slightly different from routine pathology workflows). This affordability facilitates the extensive data collection for AI‐enhanced assessment. Using colorectal adenocarcinoma as a testbed, we collected nearly 3000 images to train the deep learning algorithm, exceeding an accuracy rate of 95% in distinguishing abnormal areas from healthy epithelium in colorectal sections. This high accuracy underscores the potential of integrating AI with NOS slides for scalable, high‐throughput computational assessment of histopathology images.

## 2 Results

### 2.1 General Design of the Smart Histological Diagnosis

**FIGURE 1:**
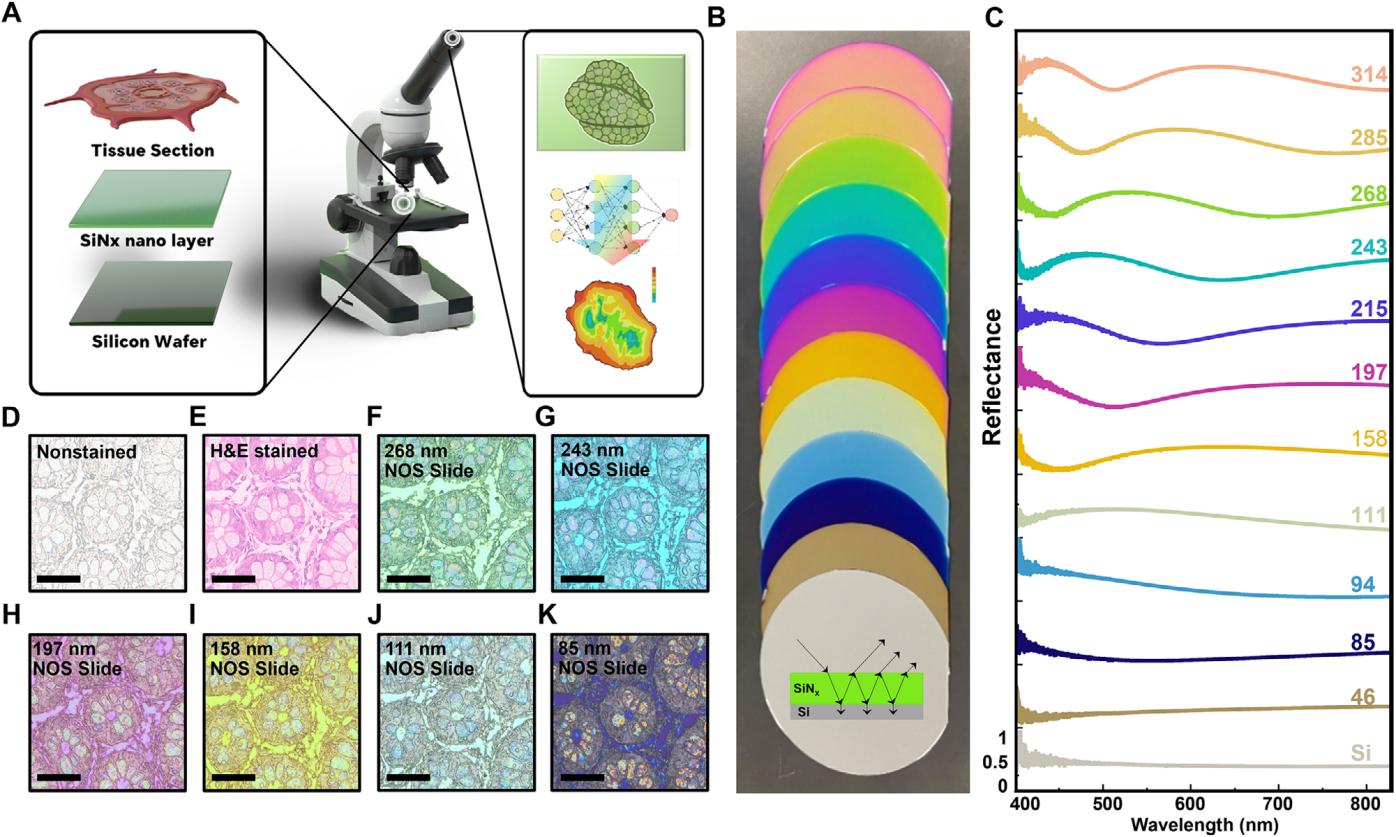
Advanced nonstained histology on structural colorimetric nanocavities‐on‐silicon (NOS) slides. (a) Schematic of smart histological diagnosis with the layered NOS slide design in the inset. (b) Optical photograph of twelve 6‐in NOS wafers, with the inset showing the optical mechanism of interference. (c) Corresponding reflectance spectra. Thicknesses of Si_3_N_4_ layers (measured in nanometers) are indicated in the top‐right corners; the bottom section illustrates the reflectance of a silicon wafer without a Si_3_N_4_ layer. Comparative microscopic images of serial nominal‐2‐µm human colorectal epithelium sections on glass slides (d) without and (e) with H&E staining. (f–k) Different colors of NOS slides, where the thickness of the top Si_3_N_4_ layer is 268 nm for (f), 243 nm for (g), 197 nm for (h), 158 nm for (i), 111 nm for (j) and 85 nm for (k). All images were captured using a 10× objective lens (NA = 0.3). Scale bars in Figure d–k: 50 µm.

Figure 1a illustrates the smart histological diagnosis system integrated with established pathological workflows, offering enhanced capabilities. The process initiates with placing a tissue section onto an NOS slide, which is designed to reveal the morphological features of the tissue via structural coloration (left panel of the inset in Figure 1a). Microscopic imaging captures vivid color and specimen detail, which is crucial for the next evaluation stage. Histopathologists can evaluate these images, or the images can be swiftly analyzed using advanced AI algorithms for high‐throughput diagnostic assessment (right panel of the inset). This approach aids a rapid and comprehensive diagnostic process, ensuring adaptability and effectiveness in contemporary medical diagnostics using the proposed strategy.

The NOS slide is a Si wafer coated with a nanometer‐thick Si_3_N_4_ layer that enables an optical thin‐film interference, as illustrated by the inset in Figure 1b. This planar nanocavity architecture is critical for the effective display of structural colors. Fine‐tuning the thickness of the Si_3_N_4_ layer, ranging from 40 to 320 nm, achieves a spectrum of vibrant colors, similar to the ultrathin Gires–Tournois etalons reported in [23]. Due to the highly lossy nanocavity structures, the selected narrowband visible light is absorbed by the Si wafer, resulting in blue, gray, yellow, purple, green, and pink colors, as depicted in Figure 1b. To reveal the physical mechanism of this vivid color change of the NOS slide, we characterized the optical reflectance of the NOS slides as shown in Figure 1c, showing noticeable shifts in resonance with varying Si_3_N_4_ thicknesses, thus underpinning the interference mechanism of the structural coloration [23, 24, 25] (see optical characterization in **Methods**). This color tunability, achieved via optical interference in reflected light [23, 24], circumvents the need for traditional labor‐intensive H&E staining or intricately fabricating nanopatterns on metallic films [17, 26]. Furthermore, to further demonstrate the superior optical feature of the proposed NOS chip, transfer‐matrix simulations were performed to compare with Fabry–Pérot and Fano‐type thin‐film models (see modeling details in Figure S1), confirming that the NOS configuration exhibits a comparable hue‐shift sensitivity while maintaining superior angular (Figure S2) and polarization robustness (Figure S3) under realistic imaging conditions.

Compared with top‐down lithographically fabricated nanopatterns using conventional Electron Beam Lithography/Focused Ion Beam milling, NOS slides have scalable manufacturability under regular laboratory conditions, as demonstrated by the 6‐in sample in Figure 1b, highlighting the cost‐effectiveness and practicality of this approach for histological applications (details in Table S1). We prepared and mounted a series of tissue sections on glass slides, including one nonstained (Figure 1d) and one H&E‐stained (Figure 1e), and prepared six NOS slides with different dielectric layer thicknesses (without staining, Figure 1f–k) to demonstrate the potential of NOS slides in colorimetric histology (see the pathology workflow in **Methods**, Figure S4 and Table S2). As observed in the microscopic images under consistent calibrated illumination (see Figure S5), the NOS slides (Figure 1f‐k) present a significantly richer color palette than those on the glass substrate (Figure 1d, e), reinforcing the capability of NOS slides to provide an essential color contrast for advanced histological examination.

### 2.2 Colorimetric Analysis on NOS Slides

**FIGURE 2:**
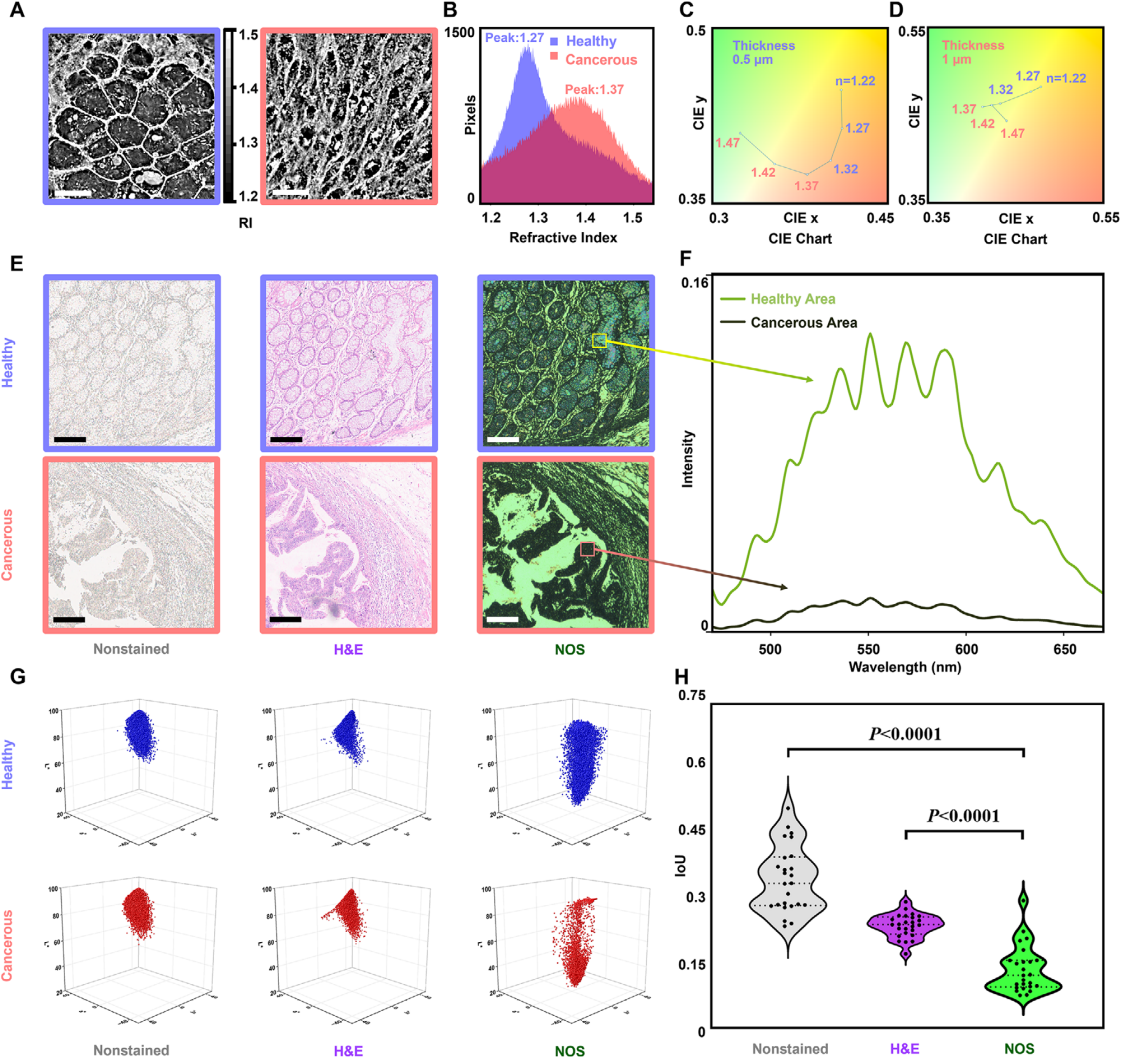
Analysis of structural colorimetric nanocavities‐on‐silicon (NOS) slides. (a) Grayscale images of refractive index (RI) distribution maps of healthy and cancerous colon tissue; grayscale values correlate with the RI. Scale bars: 10 µm. (b) Statistical distribution of the RI values of tissue in (a). (c) Simulated, 0.5 µm, *n* = 1.22–1.47 material color position change on green NOS slides on the CIE chart. (d) Simulated, 1 µm, *n* = 1.22–1.47 material color position change on green NOS slides on the CIE chart. (e) Microscopic images of human colorectal epithelium sections, highlighting healthy (blue box) and cancerous (red box) regions for calculation and analysis. All images on glass slides were scanned using the Leica Aperio scanner equipped with a 20× objective lens (NA = 0.75), whereas the two NOS images were captured using a 4× objective lens (NA = 0.13). Scale bars: 200 µm. (f) Micro‐area multispectral results of areas on green NOS slides in (e). (g) The CIELAB space of healthy (blue, upper panel) and cancerous (red, lower panel) regions. (h) Violin plots of the intersection over union (IoU) of cloud points between healthy/cancerous point clouds of samples on slides (Center dashed line indicates the median and the upper/lower dashed lines indicate the IQR; n=25 per group). Using the two‐sided independent samples t‐test, the *p*‐values are 1.94×10^‒14^ for the glass vs. NOS and 5.80×10^‒10^ for H&E vs. NOS, both significant at α=0.0001.

To elucidate the mechanisms underlying the color changes observed in tissue samples mounted on NOS slides, we analyzed how the physical properties of the tissue (i.e., refractive index and thickness) influence the reflected color [27, 28, 29, 30]. First, an optical diffraction tomography (ODT) microscope [31, 32, 33] was employed to examine the RI distribution in colorectal tissue sections (Figure 2a; see **Methods** section ‘Optical characteristics of tissue sections’). The resulting 2D RI distribution images (Figure 2b) differentiate between healthy (purple curve) and cancerous (pink curve) regions in the colon epithelium. The RI values of cancerous tissue are densely concentrated around 1.37, whereas the RI values of healthy tissue predominantly cluster around 1.27 (see ODT calibration in Figure S6). This disparity in RI values induces a distinct color change on the NOS slide, providing complementary information to traditional morphological observations. Second, AFM measurement on dewaxed nominal 2‐µm paraffin sections was performed, showing that the actual tissue thickness typically falls within 0.3–1 µm (Figure S7). Using these measured values, we then simulated the color of tissue layers with thicknesses of 500 nm (Figure 2c) and 1 µm (Figure 2d) on the green NOS slide by varying the refractive index to further explore the optical response (see Figure S8). The results show that small variations of approximately 3% in RI or thickness can lead to a just noticeable color difference perceived by the human eye (see details in Table S3), confirming that both parameters contribute jointly to the observed color change. These findings demonstrate the capability of NOS slides to sensitively detect subtle variations in both refractive index and thickness, and to translate these physical differences into discernible color contrasts within tissue sections, revealing their potential for high‐contrast, label‐free histological assessment through pathology‐relevant contrast generation.

To validate the sensitivity of this colorimetric response, we prepared a series of colorectal cancer tissue sections and mounted them on both standard glass slides (Figure 2e, left panel: non‐stained; central panel: H&E‐stained) and green NOS slides (Figure 2e, right panel). Notably, the NOS slides enhanced the contrast between cancerous (lower panel) and healthy (upper panel) regions, making morphological differences more pronounced (see control analyses in Figure S9 and Table S4, showing that this contrast enhancement was not affected by artifacts like surface glare or reduced depth averaging). We measured the microscopic reflection spectra of representative healthy and cancerous regions on the same NOS slide to quantitatively assess this visual distinction (Figure 2f; Figure S10 for technical details). The spectral analysis revealed significant brightness and spectral feature variations between the two regions, corroborating the heightened visual contrast enabled by NOS slides.

To assess the observed colorimetric differences in images quantitatively, we applied the standard CIELAB color space [34] comprising three primary channels: L* (lightness), a*, and b* (chromatic components corresponding to human color perception). This work analyzed the images from healthy and cancerous tissue pixel‐by‐pixel, mapping each pixel to a 3D CIE L*a*b* space, as illustrated in Figure 2g (see **Methods** section ‘Colorimetric analysis of tissue section’ and **Figures** S11). Notably, although a small fraction of pixels from different regions can exhibit similar CIELAB values, the diagnostically relevant separation is captured at the contiguous region level by the overall color‐distribution point clouds. Here, we introduced the intersection over union (IoU) metric, quantifying the overlap of colorimetric data clouds in the 3D space [35] to evaluate the similarity between the color distributions of healthy and cancerous tissue objectively. The IoU values range from 0 (no similarity) to 1 (identical), providing a robust measure of the spatial separation between colorimetric point clouds. Importantly, the color response after rehydration/re‐dehydration is stable and repeatable, as shown by a complementary control experiment in Figure S12. As presented in Figure 2h, in the comparison of 5x5 groups of healthy and cancerous point clouds (see Table S5), the average IoU of NOS slides is 0.13 (right column), significantly lower than the IoU of 0.34 observed for nonstained samples on glass slides (left column) and 0.24 for H&E‐stained samples (middle column). Additionally, alternative colorimetric indices, such as the Frobenius norm [36] and Chamfer distance [37], can further quantify spatial differences between the point clouds representing healthy and cancerous tissue (see Figure S13 and **Tables** S6 and S7). These results indicate that the NOS slide achieves superior color separation, enhancing the visual differentiation between healthy and cancerous tissue.

As a direct benchmark against established label‐free imaging, we additionally imaged the nonstained serial section on standard glass slides using transmission phase‐contrast and quantified the corresponding tissue regions with the same CIELAB/IoU workflow (Figure S14). Those results showed that while phase‐contrast can produce stronger intensity contrast, the NOS slide uniquely delivers an immediately interpretable structural‐color, thereby providing an imaging readout that is closer to routine color‐based pathology viewing.

Across all these quantitative comparisons, NOS slides exhibit more significant distinctions in point cloud geometry and distance metrics than both nonstained glass‐slide imaging and traditional H&E staining. These findings demonstrate the enhanced capability of NOS slides in representing colorimetric differences, reinforcing their potential for distinguishing cancerous areas in tissue sections in pathological analysis.

### 2.3 Universal Histopathological Demonstration

**FIGURE 3:**
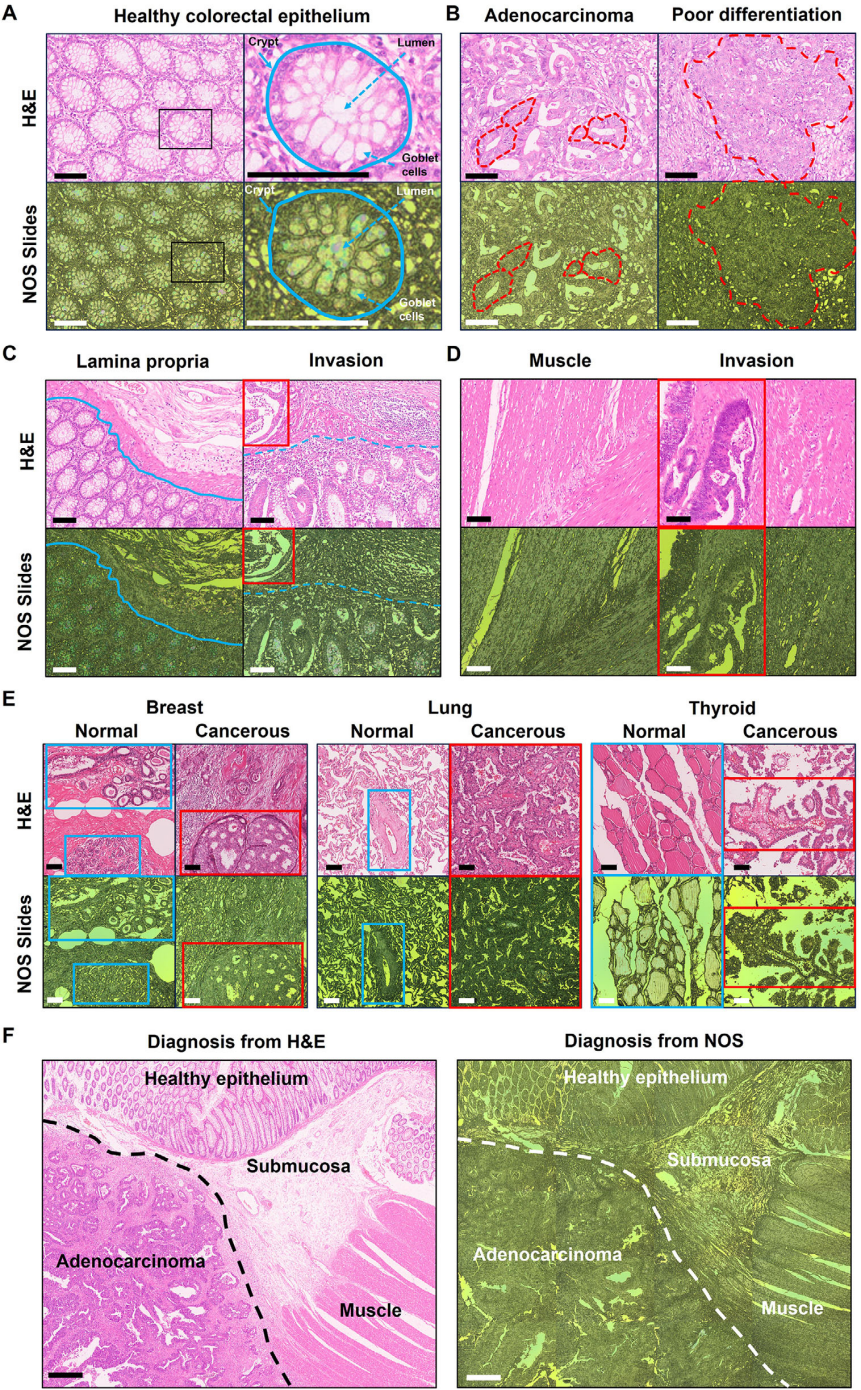
Comparison of pathological serial sections of organs and tissue in H&E‐stained and NOS slide images. (a) Healthy colorectal epithelium with a zoomed‐in epithelial cell. (b) Colorectal adenocarcinoma and a poorly differentiated tumor. (c) Healthy/cancer‐invaded colorectal lamina propria. (d) Healthy/cancer‐invaded colorectal muscle layer. (e) Healthy/cancerous breast (left panels), lung (central panel), and thyroid tissue sections (right panel). (f) A colorectal case with diagnostic results. All images on glass slides were scanned using the Leica Aperio scanner equipped with a 20× objective lens (NA = 0.75), whereas the two NOS images were captured using a 10× objective lens (NA = 0.3). Scale bars in Figure a‐e: 100 µm; in Figure f: 500 µm.

To assess the versatility of NOS slides for general pathology, we recruited 60 patients and obtained 120 tissue wax blocks (including colorectal, breast, lung, and thyroid tissue). Two serial tissue sections were prepared from each block to directly compare the H&E‐stained and NOS samples (120 for each method). We examined three representative regions in the colorectal tissue—epithelium (Figure 3a, b), lamina propria (Figure 3c), and muscle (Figure 3d)—for a detailed evaluation. As depicted in Figure 3a, the NOS slides (lower panel) faithfully recapitulated the glandular organization of healthy colorectal epithelium observed in H&E‐stained sections (upper panel). At the cellular level (right panel), NOS imaging distinctly revealed critical glandular features, including round‐to‐oval cell morphology, goblet cells, lumens, and their encapsulation in crypt structures, characterized by alternating bright blue‐green and darker green regions.

Conversely, in Figure 3b, NOS slides highlighted epithelial abnormalities in colorectal adenocarcinoma. In moderately differentiated adenocarcinoma (left panel), hallmark malignant features, such as the absence of goblet cells, cellular pleomorphism, spindle‐shaped cells, and a disrupted glandular architecture, were evident. In poorly differentiated adenocarcinoma (right panel), NOS imaging captured pronounced architectural disorganization and increased cellular atypia, with darker regions indicating significant structural alterations relative to the normal epithelium (Figure 3a).

For other colorectal tissue compartments, NOS slides provided sharp color contrasts, delineating distinct anatomical layers. In Figure 3c, the mucosa and submucosa were separated by the muscularis mucosae (blue solid lines), whereas in Figure 3d, the circular and longitudinal muscle layers were defined well. Under pathological conditions (right panels in Figure 3c, d), NOS imaging facilitated identifying adenocarcinoma invasion in the lamina propria (blue dashed lines, Figure 3c) and muscle layers (red rectangles, Figure 3d). We extended the analysis to a diverse range of human tissue, including breast, lung, and thyroid samples, to assess the versatility of NOS slides in pathology further (Figure 3e). Across all examined tissue types, NOS imaging reliably captured critical histological features (highlighted by squares in Figure 3e), closely paralleling those observed in H&E‐stained sections, and also exhibited well‐separated CIELAB color distributions between healthy and cancerous regions (Figure S15). To demonstrate the distinctive NOS coloration for pathologists, a validation with H&E‐like color mapping and reader study was performed, confirming consistent diagnostic clarity across color modes on NOS slides (Figure S16 and Table S8). These high‐contrast visualizations reinforce the feasibility of stain‐free histology of NOS slides, recapitulating key morphological features observed in matched H&E sections and supporting the feasibility of stain‐free histopathological visualization in these representative organs. To validate the diagnostic reliability of NOS slides further, we expanded the analysis to numerous colorectal cases, evaluating their potential integration into routine pathological workflows. In particular, we examined whether NOS imaging alone could provide sufficient histological information for pathologists without relying on H&E staining. As part of this assessment, we conducted a blind comparison involving 50 colorectal sections. One professional pathologist identified and labeled critical histological regions using H&E‐stained images, while a second pathologist independently performed the same task using NOS images. As depicted in **Figure 3f**, a representative case demonstrates that both pathologists independently provided identical tissue area delineations. Notably, in critical boundary regions of cancerous lesions, the NOS diagnostic group produced the same level of precision as the H&E diagnostic group, distinguishing malignant from healthy tissue. From these correctly classified regions, representative healthy–cancerous pairs were further examined in the CIELAB color space (Figure S17), revealing two well‐separated chromatic clusters with IoU values consistent with those in **Figure 2h**. A quantitative analysis confirmed the diagnostic reliability of NOS slides. Based on the statistical evaluation [38, 39, 40] of 50 colorectal cases (see ‘Statistical analysis of pathological diagnoses’ in **Methods**), the NOS diagnostic group achieved an impressive consistency rate of 99.0% and Cohen’s Kappa coefficient (κ) of 0.983 in distinguishing cancerous from healthy colorectal tissue sections, using the H&E diagnostic group assessments as the reference standard (see Table S9). This finding emphasizes the feasibility of NOS imaging as a stain‐free auxiliary method for histopathological diagnosis, enabling pathologists to extract highly accurate diagnostic information without conventional staining. Furthermore, the consistent and reproducible colorimetric information provided by NOS slides presents new opportunities for AI‐assisted histopathology, potentially streamlining diagnostic workflows and enhancing clinical pathology efficiency.

### 2.4 Artificial Intelligence‐Assisted Auxiliary Assessment

**FIGURE 4:**
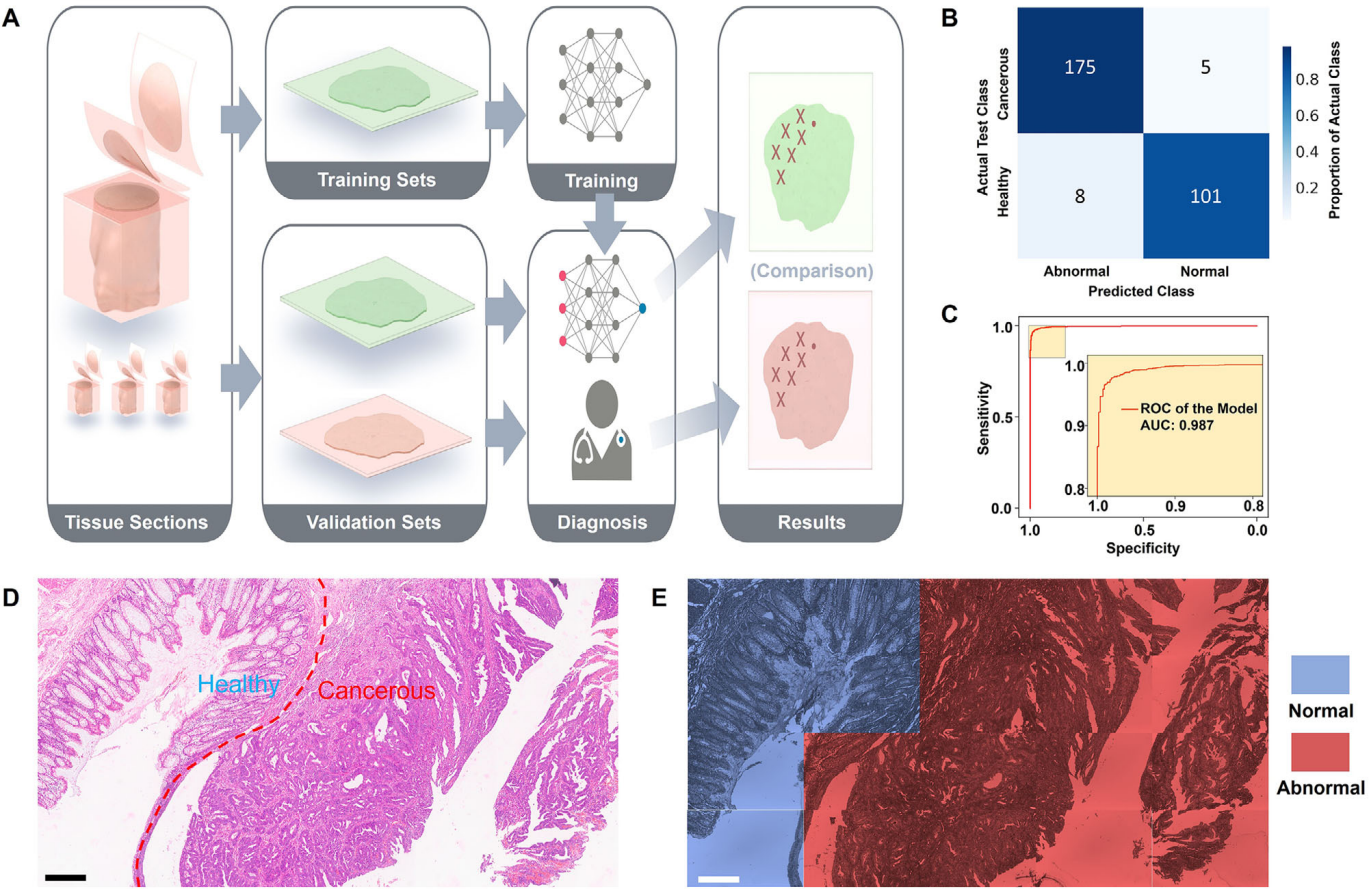
Machine learning algorithm for auxiliary assessment and results. (a) Workflow for training AI algorithms and testing auxiliary assessment performance. (b) Confusion matrix resulting from testing on the test dataset. (c) Receiver operating characteristic curve of the model, which achieved an AUC of 0.987. (d) Pathological diagnosis result for a colorectal tissue section from H&E‐stained images assessed by pathologists. (e) On a consecutive nonstained section on the NOS slide in (d), the judgment result by the DenseNet121 model. Scale bar: 500 µm.

Compared with the pioneering stain‐free histology on nanoplasmonic chips fabricated using top‐down lithography processes [17], the NOS slide facilitates collecting numerous images for training in AI‐assisted histology due to its significantly lower manufacturing costs. Exploiting this advantage, we employed an AI classification model (DenseNet121, see the model structure in Figure S18) based on convolutional neural networks (CNNs) [41]. to assist in the initial cancer diagnosis. Figure 4a depicts the overall workflow and the logic framework of the AI‐assisted auxiliary diagnosis process. In this study, the classified colorectal healthy and cancerous tissues identified on NOS slides in Figure 3 served as the experimental dataset (Table S9).

The training dataset, comprising 2651 images, was categorized into two classes: healthy epithelium (negative) and cancerous epithelium (positive). An additional 289 images were acquired as the test dataset to evaluate the diagnostic performance of DenseNet121. Both datasets were organized strictly at the patient level to ensure that no slides nor image tiles from the same patient appeared in both the training and testing sets, thereby preventing data leakage and ensuring a fair and reliable model evaluation (see details in Table S10). Owing to the well‐defined contrast between healthy and cancerous epithelial structures on NOS slides, the model achieved a maximum training accuracy of 98.6% with consistently low training loss, demonstrating rapid convergence (see Figure S19). The confusion matrix for the test dataset (Figure 4b) affirmed the model's strong performance, achieving an accuracy of 0.955, along with high precision (0.9563), recall (0.9722), F1 score (0.9642), and Cohen's κ value (0.9037), highlighting its robustness in distinguishing abnormalities within colorectal epithelium (see **Methods** section ‘Performance Indicators’). Model efficacy was further corroborated by the receiver operating characteristic curve in Figure 4c, with an Area Under the Curve value of 0.987, indicating excellent classification capability. To further interpret the model's performance and ensure its reliability, extra evaluation and interpretability analyses, encompassing confusion patterns, calibration (reliability) diagrams, and representative failure exemplars, were conducted, supporting the robustness of the trained model (Figure S20). Moreover, evaluation using an alternative CNN architecture, ResNet50 [42], on the same dataset yielded comparable results (see Table S11). A subsequent cross‐validation analysis using another patient‐level partition to train a new DenseNet121 model yielded slightly lower yet comparable performance metrics (**Tables** S12 and S13). Collectively, these results underscore the promise of deep learning models as reliable and efficient tools to support pathologists in the image‐based assessment and screening of colorectal cancer on the NOS slides.

To further assess the classification performance of the trained algorithms, we compared the region‐level annotations from H&E‐stained tissue performed by pathologists with those generated by the trained model. As displayed in Figure 4d, we employed a 10× objective lens to capture H&E‐stained images and assembled a large‐area image with total dimensions of 7.16 × 3.81 mm (27.3 mm^2^). The pathologist identified and annotated two distinct regions corresponding to typical features of adenocarcinoma and healthy epithelium. We attached an adjacent tissue slice to the NOS slide and captured the nonstained images of the identical region. As illustrated in Figure 4e, we applied unique color codes to label these regions using the DenseNet121 model. Notably, the AI model accurately segmented these regions, producing results closely aligned with the pathologist's annotations in Figure 4d. Importantly, the model also maintained consistent classification results under a different illumination intensity (Figure S21), demonstrating its robustness against illumination variations. This high concordance underscores the capability of the model to segment NOS slide images comparably to manual delineations of H&E‐stained tissue performed by expert pathologists. Moreover, in a computational environment equipped with a single Nvidia A100 80 G GPU, the algorithm required only 0.5 s to generate each output image (i.e., the color‐labeled Figure 4e took approximately 10 s in total). This rapid and reliable processing capability demonstrates the potential of AI‐assisted tools to accelerate large‐scale image review and computational triage of histological images on the NOS slides.

## 3 Conclusion

In summary, this study presents cost‐effective NOS technology, applying an 80‐270 nm‐thick SiNx film on silicon wafers to overcome cost barriers associated with advanced nanoplasmonic chips. Via optical interference to generate structural colors, NOS slides eliminate the need for conventional histological stains, streamlining tissue visualization and supporting faster image acquisition and review workflows. The NOS slides demonstrate comparable effectiveness in revealing morphological features and color contrasts of organ tissue sections under standard microscopy, validated against conventional H&E staining. This approach offers a scalable alternative to traditional staining, addressing labor intensity, variability, and cost challenges. Notably, the affordability of NOS slides facilitates seamless integration with AI image‐based assessment, a capability not previously demonstrated with nanoplasmonic technologies. In this proof‐of‐concept study, an AI model trained on a dataset of over 3000 NOS slide images of colorectal cancer tissue achieved >96% accuracy in distinguishing cancerous from healthy tissue. This initial success highlights the technical feasibility and potential of NOS slides for high‐throughput, rapid, AI‐assisted screening and decision support. By combining nanotechnology, optics, and AI, NOS slides provide a transformative solution for digital pathology, precision medicine, and sustainable laboratory practices. Their cost‐effectiveness and scalability make them suitable for resource‐limited settings while reducing environmental and health hazards associated with chemical staining. This technology has considerable promise for transforming histopathology workflows, enabling scalable stain‐free imaging and computational analysis, and accelerating access to pathology services, positioning NOS slides at the forefront of the rapidly evolving digital diagnostic market.

## 4 Methods

### 4.1 Preparation of NOS Slides

Plasma‐enhanced Chemical Vapor Deposition was employed to deposit Si_3_N_4_ layers on an as‐received, single‐sided polished 6‐in Si wafer, using a precursor gas mixture of silane, ammonia, and nitrogen under low‐pressure plasma conditions (More details in Table S1). Reproducibility was ensured primarily by optical characterization of the deposited film (See details below). The deposited Si wafer was cut into 25 × 75 mm slides (the same size as ordinary medical glass sheets) with a Ytterbium Fiber Laser (Thorlabs). Then, the surface was ultrasonically cleaned with isopropyl alcohol and water before obtaining the clean NOS slides. vAfter dicing/cleaning, the optical response and microscope‐level color uniformity were re‐verified to confirm that post‐processing did not alter the structural color and that no residual debris remained. No extra silanization or surface chemistry was performed.

### 4.2 Optical characterization of NOS slides

SiN_x_ film thickness was first verified by ellipsometry across each wafer to confirm the thickness with accuracy. The reflectance spectra of the NOS slides were measured using a Fourier transform infrared spectrometer (Bruker) equipped with a microscope (HYPERION II), within the wavelength range of 400–830 nm. Data processing and visualization of reflectance spectra were conducted using Origin 2024b.

### 4.3 Pathology Workflow

The sample preparation procedure used in this work was slightly different from the routine pathology workflow [2]: Tissue wax blocks were obtained from the sample bank of the Department of Pathology at Huashan Hospital (Shanghai, China), under the protocol number HIM‐2024‐0350. Typically, tissues were trimmed into appropriately sized blocks (e.g., 1 × 1 × 0.5 cm). Initial fixation was performed using formaldehyde, followed by dehydration through a controlled water‐ethanol gradient. The tissues were then cleared in xylene and embedded in paraffin via a xylene–paraffin gradient, using the Leica HistoCore PELORIS 3 Premium Tissue Processing System. Subsequently, the tissues were embedded in molten paraffin within molds, which were then cooled to form solid paraffin blocks. The paraffin blocks were sectioned into 2‐µm‐thick serial slices using a Leica microtome. Sections were mounted onto glass or NOS (non‐coated substrate) slides, dewaxed with xylene, rinsed through a graded ethanol‐water series, and subsequently maintained in an air‐dried state, which was sometimes considered in the context of long‐term archival suitability. However, in this study, since the structural‐color readout relies on the intrinsic optical environment of air‐dried tissue sections, liquids (including mounting media) were avoided, as they can reduce structural‐color contrast. Samples on glass slides requiring histological analysis were stained with hematoxylin and eosin (H&E) and mounted using a standard slide mounting medium. In contrast, sections on NOS slides, as well as nonstained sections on glass slides, were not subjected to any additional processing. Further details are provided in Figure S4 and Table S2. The cellular‐scale registration accuracy between serial H&E and NOS sections is shown in Figure S22.

### 4.4 Image Collection of Tissue Sections

Image data from tissue sections mounted on standard glass slides, including both H&E‐stained and nonstained sections, whole‐slide image acquisition was performed using an automated slide scanner (Leica Aperio GT 450) in transmitted‐light brightfield mode. For the tissue sections mounted on NOS slides, image data were acquired using a reflection microscope (Nikon Eclipse Ti2) equipped with an automated scanning stage and automated stitching software. Whole‐section mosaics on NOS slides were generated by automated tile scanning across the tissue region with fixed illumination and camera settings within each scan, followed by software‐based stitching without manual montage (See the automated processing in **Supplementary Video** S1). Transmission phase‐contrast images of nonstained serial sections on glass slides were acquired on the same Nikon microscope using the corresponding phase‐contrast configuration.

### 4.5 Optical Characteristics of Tissue Sections

#### 4.5.1 Refractive Index (RI) Distribution of Tissue Sections

The RI distribution of tissue sections was measured using an optical diffraction tomography (ODT) microscope (MH‐HoliView, Pellucid Optics Technology (Nantong) Co., Ltd.), operated in conjunction with the MH‐IntellyAcq image processing software. A 40× objective lens was employed to collect scattering signals from the sample, enabling reconstruction of the RI map.

#### 4.5.2 Color Simulation of Tissue Sections on NOS Slide

The reflection spectra R(λ) of the NOS slides (Si/Si_3_N_4_/Tissue) were numerically simulated using the transfer matrix method, with spectral calculations performed at 1 nm resolution across the 360–830 nm wavelength range [43]. To quantify the perceived color characteristics, the computed reflection spectra were converted to CIE 1931 color coordinates (x, y) through integration with the standard observer color‐matching functions, which mathematically represent human chromatic perception:

(1) $$X=\int R\left(\lambda \right)\bar{x}\left(\lambda \right)d\lambda$$

(2) $$Y=\int R\left(\lambda \right)\bar{y}\left(\lambda \right)d\lambda$$

(3) $$Z=\int R\left(\lambda \right)\bar{z}\left(\lambda \right)d\lambda$$

The resulting chromaticity coordinates (x, y) were then determined by normalizing these tristimulus values:

(4) $$x=\frac{X}{X+Y+Z}$$

(5) $$y=\frac{Y}{X+Y+Z}$$

The points of the obtained chromaticity coordinates (x, y) were drawn on the CIE chart by using MATLAB.

#### 4.5.3 Thickness Measurement of Tissue Sections

The thickness distribution of tissue sections was characterized using an atomic force microscope (AFM, Bruker, Dimension Icon SPM) to assess the surface variation of the samples. All measurements were performed in a dry state without the addition of any liquid medium. A FESPA‐V2 model AFM probe was employed, providing a nominal tip radius of about 10 nm and a spring constant of approximately 2.8 N/m. All measurement data were automatically recorded by the integrated software, and subsequent data processing and visualization were conducted using Origin 2024b. Detailed results are provided in Figure S7.

#### 4.5.4 Reflectance Spectra of Tissue Sections on NOS Slide

Reflectance spectra of tissue sections mounted on NOS slides were acquired using a Leica TCS SP8 confocal microscope. Spectral data in Figure 2F were collected with a 5 nm bandwidth across excitation wavelengths ranging from 470 to 670 nm. More details about the setup were listed in Figure S10.

### 4.6 Data Analysis From Images of Tissue Section

#### 4.6.1 CIELAB Color Space

The CIELAB color space (Figure S11) established by the International Commission on Illumination (CIE) in 1976, represents colors through three parameters: L* (lightness), a*, and b*. The L* value quantifies perceptual lightness, ranging from 0 (black) to 100 (white). The a* axis captures the green–red opponency, with negative values indicating green and positive values indicating red. Similarly, the b* axis denotes the blue–yellow opponency, with negative values corresponding to blue and positive to yellow. In this analysis, the a* and b* values were clamped within the range of −128 to 127. For color space transformation, a square image region of 81 × 81 pixels (6561 pixels in total) was selected. The MATLAB function rgb2lab was employed to convert RGB pixel values into the corresponding CIELAB components (L*, a*, b*), yielding a 3D dataset for each pixel. These triplet values were subsequently visualized as a 3D point cloud.

#### 4.6.2 Intersection Over UNION (IoU)

To achieve a more accurate assessment of spatial similarity between datasets, we employed a kernel density estimation‐based Intersection over Union metric (3D KDE‐IoU) [35]. This method involves three principal stages: kernel density estimation (KDE), numerical grid discretization, and density‐based IoU computation. Given two 3D datasets, KDE was first applied to estimate their continuous probability density functions *p_A_* (*x*) and *p_B_* (*x*). This was accomplished by convolving the discrete data points with a 3D Gaussian kernel, where the bandwidth parameter governs the extent of smoothing. The resulting continuous density estimates werethen evaluated over a uniformly sampled 3D grid that spans the combined spatial domain of the datasets. This discretized grid enables numerical integration by computing KDE values at each grid point.

The KDE‐IoU metric was then derived by calculating the voxel‐wise minimum and maximum of the estimated densities. Specifically, the intersection was obtained by summing the pointwise minimum of *p_A_* (*x*)and *p_B_* (*x*) across the grid domain Ω, while the union was the sum of the pointwise maximum:

(6) $$IoU_{KDE}=\frac{\sum \Omega \min\left(p_{A}\left(x\right),p_{B}\left(\left.x\right)\right)\right.}{\sum \Omega \max\left(p_{A}\left(x\right),p_{B}\left(\left.x\right)\right)\right.}$$

This ratio yields a similarity score that captures both spatial overlap and distributional alignment between the datasets.

#### 4.6.3 Frobenius Norm (F‐Norm)

The Frobenius norm was a fundamental metric for quantifying the magnitude of higher‐dimensional arrays [30], with broad applicability across disciplines such as optics, computer vision, and machine learning. It enables the reduction of complex, multi‐dimensional structures to a single scalar value, thereby facilitating global comparisons between datasets. In the context of 3D point clouds, the Frobenius norm effectively captures the cumulative discrepancy between corresponding elements of two datasets. For two 3D point clouds, A and B, represented as matrices or tensors of equal dimensions, the F‐norm of their element‐wise difference was computed as:

(7) $$A-B_{F}=\sqrt{\sum_{i=1}^{M}\sum_{j=1}^{N}\sum_{k=1}^{P}{\left|A_{ijk}-B_{ijk}\right|}^{2}}$$

where L, M, and P denote the dimensions of the 3D arrays. This formulation sums the squared differences across all corresponding elements, and the square root of this total yields the Frobenius norm. A higher Frobenius norm indicates a greater overall deviation between the two point clouds, reflecting substantial structural or distributional differences. In this study, the Frobenius norm was used as a complementary measure to assess global alignment in addition to the IoU metric.

#### 4.6.4 Chamfer Distance

The Chamfer distance was a widely adopted metric for quantifying the geometric similarity between two sets of 3D points [31]. It measures the average closest‐point distance between point clouds, thereby capturing both global alignment and fine‐grained structural detail. To ensure equal contribution from all spatial dimensions, each coordinate axis was first linearly normalized to the [0,1] interval. Subsequently, the Euclidean distance from each point in point cloud A to its nearest neighbor in point cloud B, and vice versa, was computed using MATLAB's pdist2 function. To accommodate application‐specific considerations such as regional saliency or non‐uniform point density, optional weighting schemes were applied to the nearest‐neighbor distances prior to averaging. The bidirectional average of these distances yields a single scalar value that encapsulates the overall similarity between the two point sets. Finally, the resulting Chamfer distance was rescaled back to the original physical units to maintain consistency with the data's native spatial resolution. All computational procedures were conducted in MATLAB, with subsequent analyses performed using GraphPad Prism (version 9.0.2).

### 4.7 Statistical Analysis of Pathological Diagnoses

#### 4.7.1 Colorectal Sections Cohort

A total of 50 colorectal tissue sections were included in this analysis. Each case was represented by two types of histological preparation: non‐stained NOS slides and conventional H&E slides. The 50 NOS and 50 H&E cases were independently assigned to two separate groups of pathologists for blind evaluation. Each NOS image spanned an area of approximately 1.42 mm × 0.95 mm, with each case yielding between 100 to 200 images. For each NOS image, pathologists in the NOS group were asked to make one or more of the following categorical determinations: (i) healthy epithelium, (ii) cancerous epithelium, and (iii) non‐epithelial tissue, which encompassed structures such as stroma, muscularis propria, and serosal layers. Each image received at least one determination. To assess diagnostic consistency, the classifications made on NOS images were compared with those made on corresponding regions in the H&E slides, which served as the reference standard. Inter‐operator agreement between the NOS and H&E evaluations was quantified using Cohen's Kappa statistics (as explained below). All statistical analyses were conducted using IBM SPSS Statistics (version 25.0). Additional tabulated results are provided in Table S9.

#### 4.7.2 Cohen's Kappa Coefficient (κ)

**κ** is a robust statistical metric used to quantify the degree of agreement between two raters or classification methods [38, 39], while correcting for agreement that may occur by chance. In this study, κ was computed based on a three‐class confusion matrix derived from categorical evaluations provided by two diagnostic approaches. The observed agreement rate, P_0_, was first calculated using the diagonal elements of the confusion matrix:

(8) $$P_{0}=\frac{n_{11}+n_{22}+n_{33}}{N}$$

where n_ii_ denotes the number of instances in which both methods assigned the same class i, and N represents the total number of observations. The expected agreement by chance, P_e_, was computed as:

(9) $$P_{e}=\sum_{k=1}^{3}\frac{r_{k\cdot }c_{\cdot k}}{N^{2}}$$

where r_k_ and c_k_ denote the row and column marginal totals, respectively, for class k. The final Kappa coefficient was then derived using the standard formulation:

(10) $$\kappa =\frac{P_{o}-P_{e}}{1-P_{e}}$$

This coefficient yields a value between −1 and 1, where 1 indicates perfect agreement, 0 indicates agreement equivalent to chance, and negative values indicate systematic disagreement. All computations were performed using IBM SPSS Statistics (version 25.0).

### 4.8 Performance Indicators of AI Models

The following metrics were used to evaluate the AI models in this work:

#### 4.8.1 True Positive (TP)

Assigned when the model classified a test image as positive, and the corresponding region was identified as cancerous epithelium by pathologists based on the adjacent H&E‐stained section.

#### 4.8.2 False Positive (FP)

Assigned when the model classified a test image as positive, while the corresponding region was identified as healthy epithelium by pathologists based on the adjacent H&E‐stained section.

#### 4.8.3 True Negative (TN)

Assigned when the model classified a test image as negative, and the corresponding region was identified as healthy epithelium by pathologists based on the adjacent H&E‐stained section.

#### 4.8.4 False Negative (FN)

Assigned when the model classified a test image as negative, while the corresponding region was identified as cancerous epithelium by pathologists based on the adjacent H&E‐stained section.

#### 4.8.5 Accuracy

The proportion of correct predictions among the total number of cases examined.

(11) $$Accuracy=\frac{TP+TN}{TP+FN+FP+TN}$$

#### 4.8.6 Precision

Precision was the ratio of true positive predictions to the total positive predictions. It measures the accuracy of positive predictions made by the model.

(12) $$Precision=\frac{TP}{TP+FP}$$

#### 4.8.7 Recall

It was also known as sensitivity, which was the ratio of true positive predictions to the total actual positive cases. It measures the model's ability to find all positive instances.

(13) $$Recall=\frac{TP}{TP+FN}$$

#### 4.8.8 F1‐score

The F1‐score was the harmonic mean of precision and recall, providing a single score that balances both metrics.

(14) $$F1=\frac{2}{\frac{1}{precision}+\frac{1}{recall}}=2\cdot \frac{precision\cdot recall}{precision+recall}$$

#### 4.8.9 Receiver Operating Characteristic (ROC) Curve

The ROC curve visually depicts a classifier's discrimination power across all decision thresholds. To construct it, apply the trained model to the test set to obtain the predicted probability for the positive class of each sample; then sweep the threshold from 0 to 1, calculate the corresponding true‐positive and false‐positive rates, and plot these pairs.

#### 4.8.10 Area Under the Curve (AUC)

The AUC quantifies the ROC curve by measuring the area beneath it, thereby providing a single, threshold‐independent metric that summarizes overall classification performance [40]. It was defined as:

(15) $$AUC=\int_{0}^{1}TPR\left(FPR^{-1}\left(t\right)\right)dt$$

where TPR was the true positive rate, and FPR was the false positive rate. AUC values range from 0 to 1, where 1 indicates perfect discrimination, and 0.5 represents performance equivalent to random chance.

## Conflicts of Interest

The authors declare no conflicts of interest.

## Supporting information




**Supporting File 1**: advs74341‐sup‐0001‐SuppMat.pdf.

**Supporting File 2**: advs74341‐sup‐0002‐VideoS1.mp4.

## Data Availability

The data that support the findings of this study are available from the corresponding author upon reasonable request.
