# Supplementary material for: Intelligent Stain‐Free Histology on Structural Colorimetric Nanocavities
Source: Adv Sci (Weinh). 2026 Feb 12;13(22):e14340. doi: 10.1002/advs.202514340 (PMC13088267; doi:10.1002/advs.202514340)
Supplement: Supplementary file 1 — Supporting File 1: advs74341‐sup‐0001‐SuppMat.pdf. [file ADVS-13-e14340-s002.pdf]

Supporting Information for

Intelligent stain-free histology on structural colorimetric nanocavities

Qizhe Chen[1,*], Yifei Ren[2,*], Lijie Hu[3,*], Yanyan Li[4,*], Wenyue Liang[1], Jin Wang[1], Han Gao[1], Xinhai Wang[3], Jiajun Li[1], Qiutao He[2], Yingfeng Zhu[2], Haifeng Hu[5], Qiwen Zhan[5], Imed Gallouzi[4,7], Jasmeen Merzaban[4,7], Di Wang[3], Zunguo Du[2, b)], Xiaodong Gu[6, a)], Qiaoqiang Gan[1,8,c)]

[a-c)] Corresponding author:

[a)] guxiaodong@fudan.edu.cn;

[b)] duzunguo@fudan.edu.cn;

[c)] qiaoqiang.gan@kaust.edu.sa

This PDF file includes:

Figures S1 to S22

Tables S1 to S13

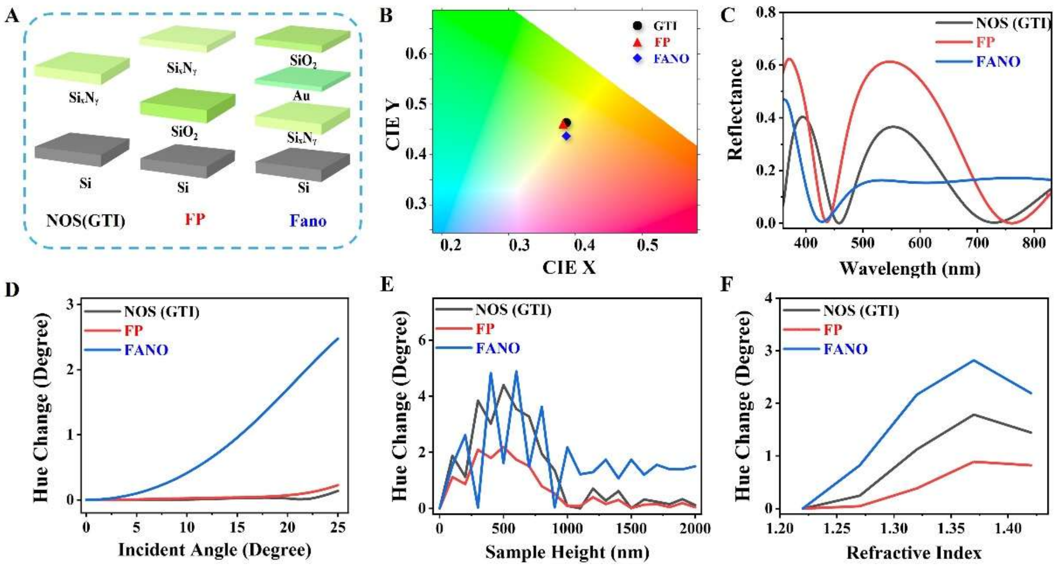

Figure S1. Transfer-matrix method (TMM) simulations comparing the optical responses of our NOS slides with representative Fabry–Pérot (FP) and Fano-type structures designed for similar green hues. (a) Schematic designs of three types of nanostructures. (b) Simulated color of three nanostructures designs on CIE chart. (c) The simulated reflectance spectra of the three cavity designs without any overlying sample under normal incidence, as plotted on the CIE chromaticity chart. (d) Angular robustness: Simulated hue variation as a function of incident angle for a sample layer with a thickness of 1 μm and refractive index of 1.32. (e) Thickness sensitivity (Δt): Hue shift as a function of sample thickness under normal incidence at a fixed refractive index of 1.32. (f) Refractive-index sensitivity (Δn): Hue shift as a function of sample refractive index under normal incidence at a fixed thickness of 1 μm.

Note: Our NOS structure is intrinsically a Gires–Tournois interferometer (GTI)–type cavity, consisting of a high-index dielectric layer (Si_xN_γ) deposited on a Si substrate, in line with canonical GTI designs reported in the previous literature (e.g., *Nature Materials* **2013**, *12(1)*, 20-24 and our own work, *Advanced Materials **2014**, 26(17), 2737–2743*).

We constructed two representative benchmarks to compare with our GTI cavity: i.e., a *Fabry-Pérot* (FP) cavity and a Fano-type thin-film structure. The structural layouts are shown in **Figure S1a**. Specifically, the FP model adopts a Si_xN_γ -SiO_2 -Si stack, (with a Si_xN_γ layer thickness of 66 nm and a SiO_2 spacer thickness of 285 nm), representing a classical two-mirror etalon that produces periodic interference fringes. The Fano model follows a design analogous to the "buried Au" architecture reported in the reference (*Reviews of Modern Physics* **2010**, *82(3)*, 2257-2298), consisting of SiO_2 (75 nm) - Au (5 nm) -Si_xN_γ (175 nm) on Si. This structure supports a narrow leaky mode coupled to a broad continuum, producing the characteristic asymmetric Fano line shape. To validate that the Fano model was correctly constructed, we fitted the simulated spectrum to the standard Fano profile (*Nature Nanotechnology* **2021**, *16(4)*, 440-446) and extracted the asymmetry parameter $\boldsymbol{q}$. The result ($\boldsymbol{q}$ = - 0.57) quantitatively confirms the presence of a Fano-type resonance. Across all models, the design parameters were

deliberately selected such that the overall hue is comparable to that of the GTI configuration, ensuring a fair basis for benchmarking. The calculated color coordinates are summarized in **Figure S1b**, showing that the GTI, FP, and Fano all map to a similar yellow-green hue region. The corresponding reflectance spectra are plotted in **Figure S1c.**

Building on these validated models, we then performed the compact TMM sweeps suggested by the reviewer to quantify sensitivity to sample thickness (Δt), refractive index (Δn), and angular variations. The results indicate that under equivalent perturbations in sample thickness (Δt), refractive index (Δn), and incident angle, the hue shifts produced by the NOS, FP, and Fano cavities remain within the same order of magnitude (**Figures S1d–S1f**). Specifically, within an incident-angle range of 0–25° (corresponding to the numerical aperture of the objective lens used in our experiment, i.e., NA = 0.4), the hue deviations in all three cavity types remain ≤ 1°, demonstrating comparable angular robustness and minimal chromatic distortion under realistic reflection-microscopy conditions. When the sample thickness varies between 0 and 2 μm, the resulting hue shifts for all three structures are typically in the range of 2–5°, while refractive-index variations between 1.22 and 1.42 produce hue changes of roughly 2°. These trends collectively illustrate that each cavity architecture can sensitively and reproducibly encode the physical properties of the sample (thickness and refractive index) into discernible structural-color variations, while maintaining high stability against angular perturbations.

In summary, these results emphasize that the NOS configuration provides an optimal balance between chromatic sensitivity and structural simplicity, ensuring both robust imaging performance and scalability for cost-effective, large-area NOS slide fabrication.

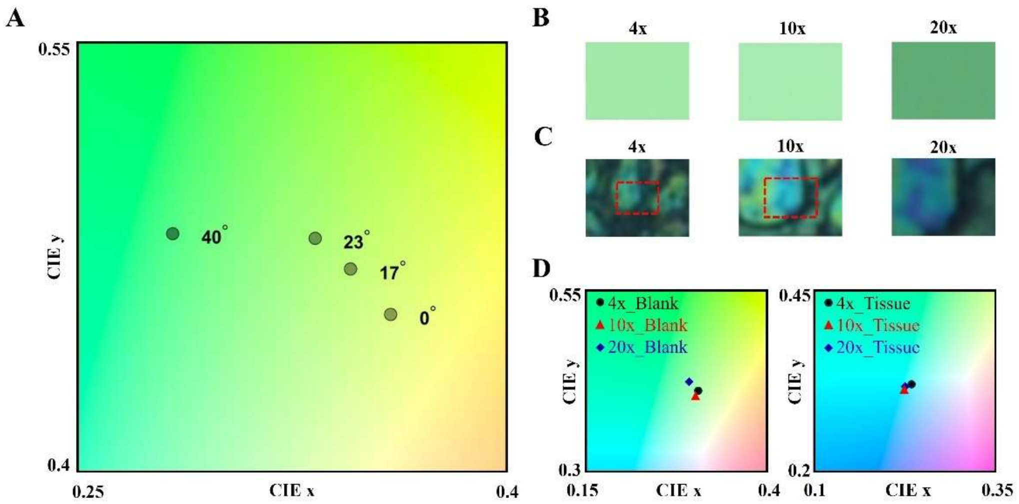

Figure S2. Validation of the influence of collection angle (objective NA) on the colorimetric response of NOS slides. (a) Simulated, n=1.33, thickness= 200 µm material color position change on green NOS slides on the CIE chart, under incident angles of 0°, 17°, 23°, and 40°. (b) The blank region of the NOS slide imaged under identical illumination conditions using 4× (NA = 0.13), 10× (NA = 0.30), and 20× (NA = 0.45) objectives. (c) The tissue section region of the NOS slide imaged under identical illumination conditions using 4× (NA = 0.13), 10× (NA = 0.30), and 20× (NA = 0.45) objectives. The red dashed box in the 4× image indicates the area corresponding to the 10× image, and the red dashed box in the 10× image indicates the area corresponding to the 20× image, demonstrating that the color comparison was made on the same region. (d) Left: The color positions of the blank NOS slide region under the three objectives plotted on the CIE chart; Right: The color positions of the tissue central region (at the same location after alignment) under the three objectives plotted on the CIE chart.

Note: In regular imaging tasks under clinical settings, the NA of objectives (4x to 20x) is usually in the range of 0.13-0.45, corresponding to the collection angle of 7°-23°. This could in principle lead to modest spectral shifts, for example slight changes in hue or saturation due to averaging over a broader distribution of incidence angles. As shown in our simulation (**Figure S2a**), the resulting changes are also small, compared with the dominant contrast mechanisms governed by tissue thickness and effective refractive index differences. We further validated this experimentally using one representative NOS slide and one representative tissue region for color comparison. Images were acquired under three objectives: 4× (NA = 0.13), 10× (NA = 0.30), and 20× (NA = 0.45), as shown in **Figure S2b** and **Figure S2c**. By comparing the hue values at the same spatial locations, the hue differences observed across these NA values (**Figure S2d)** were minimal for both the bare NOS slide and the tissue-covered region, confirming that wide-NA collection introduces negligible color variation. These results indicate that the colorimetric response of NOS slides is highly robust to moderate changes in collection angle, and the normal-incidence assumption used in simulations remains valid for typical objective configurations in histopathological imaging.

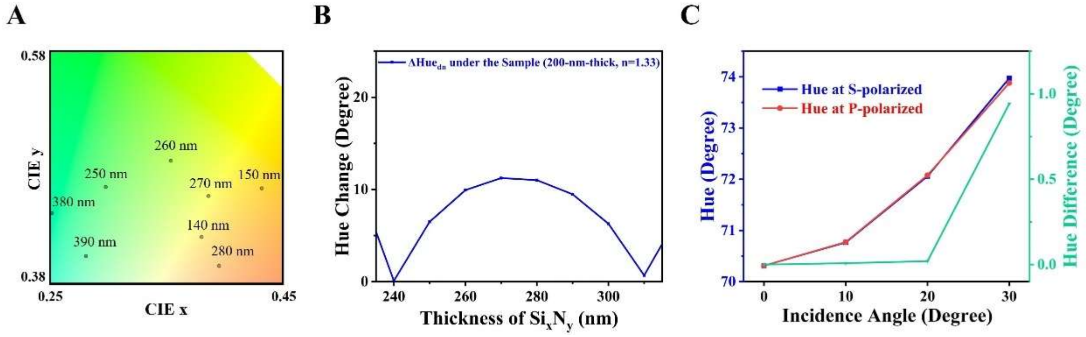

Figure S3. Design optimization and robustness evaluation of the Si_xN_γ layer thickness in NOS slides. (a) Simulated colors of blank NOS slides with Si_xN_γ thickness ranging from 0 to 500 nm (10 nm interval), plotted on the CIE chart, with the 260–270 nm region enlarged to highlight the yellow-green hue window. (b) Simulated hue difference (ΔHUE) between the baseline (200 nm, n = 1.33) and perturbed (Δn = 0.01) samples, plotted as a function of Si_xN_γ thickness within the near-270 nm range. (c) Simulated hue response of the optimized NOS structure under varying incidence angles (0–30°) for S- (blue line) and P-polarized (red line) illumination, together with their hue difference (green line).

Note: Our Si_xN_γ thickness design was developed through three stages: identifying the approximate color window, optimizing the precise thickness, and verifying robustness. Specifically:

We first determined the color window. The design goal was a yellow–green appearance, which enables direct comparison with the Nature paper (*Nature* **2021**, *598(7879)*, 65-71.) and benefits from the high visual sensitivity of the human eye in this spectral range. To this end, we performed TMM scans of Si_xN_γ thickness from 0 to 400 nm under normal incidence and mapped the calculated reflectance colors onto the CIE diagram (**Fig. S3a**). This revealed a continuous yellow–green window at t_Si_xN_γ ≈ 260-270 nm, within which the hue remained stable and vivid.

Within this window, we then sought the most effective thickness. We simulated the hue response to small variations in sample optical parameters, aiming to identify the configuration most sensitive to changes in tissue refractive index. Specifically, we set the sample thickness to 200 nm with a refractive index of 1.33 as the baseline, and then introduced a small refractive index perturbation (Δn = 0.01) before recalculating the hue. By plotting the HUE difference between the perturbed and unperturbed conditions as a function of Si_xN_γ thickness, we observed that the sensitivity peaked near 270 nm (**Fig. S3b**). This thickness was therefore selected as a practical operating point, as it provides high hue sensitivity while remaining tolerant to realistic fabrication variations.

Finally, we assessed the robustness of the designed structure with respect to both incidence angle and polarization. The results are summarized in **Fig. S3c**, where the horizontal axis denotes the incidence angle and the vertical axis represents the hue variation. The blue curve corresponds to S-polarized illumination and shows that, within the 0-30° incidence range (corresponding to the typical collection cone of the objectives used in pathology), the hue shift remains below 5°, confirming angular robustness. The red curve represents P-polarized

illumination and exhibits a nearly identical trend, indicating that the hue response is not sensitive to polarization state. In addition, the green curve compares the HUE difference between S and P polarizations at identical incidence angles, again showing only minimal deviations. Taken together, these results demonstrate that the coloration of the NOS slide is highly stable against realistic variations in both incidence angle and polarization, further validating the predictability and robustness of the design under practical imaging conditions.

In summary, the optimal Si_xN_Y thickness is approximately 270 nm. In our fabrication, the deposited layer was 269 nm, which is very close to this theoretical optimum. This choice provides high visual contrast, predictable hue behavior, and robustness against incidence angle and polarization, making it a reliable and reproducible design for NOS slides.

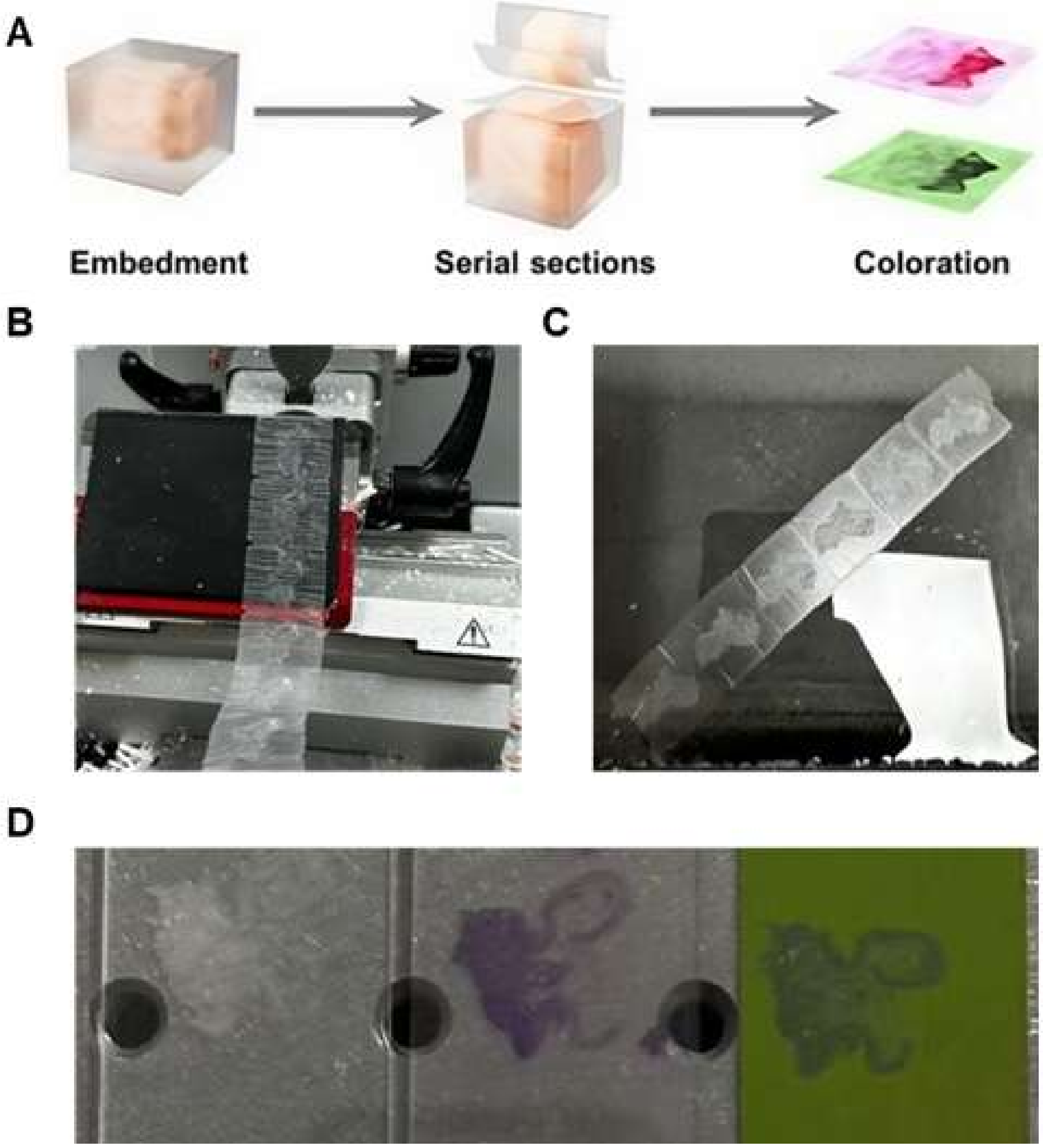

Figure S4. Pathology workflow for serial sections. (a) Schematic illustration of the pathology workflow. (b) Serial slices being obtained from a paraffin tissue block using a microtome. (c) Spreading of these slices in warm water. (d) A batch of serial tissue sections: one non-stained section on a glass slide, one H&E-stained section on a glass slide, and one nonstained section placed on a NOS slide, each prepared for further processing as detailed in **Table S2**.

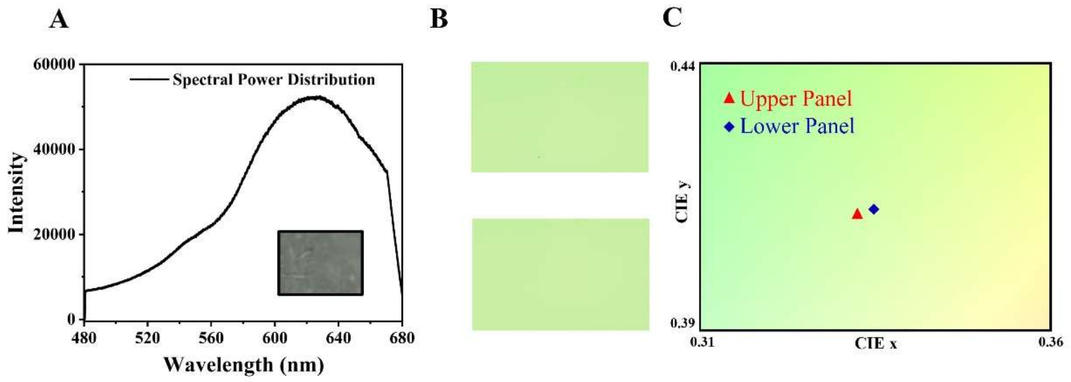

Figure S5. Verification of illumination calibration and cross-session color stability in NOS imaging. (a) Spectral power distribution of the illumination source on our brightfield microscope, measured by a spectrometer within the 470–670 nm range. The inset shows the image of the white reference tile after white balance. (b) Background images of the same NOS slide acquired in two independent imaging sessions under identical illumination and optical settings (objective lens:10x and NA=0.3). (c) Corresponding plots on CIE Chart of the background colors shown in (b).

Note: For our reflection-mode bright-field microscope, we measured the spectral power distribution (SPD) of the light source using a spectrometer within the 470–670 nm range, as shown in **Figure S5**. In addition, the inset of **Figure S5a** shows the image of the white reference tile after white balance (we used this white reference tile across all sessions). Following the procedure reported by (*IEEE transactions on image processing* **2011**, *20(9)*, 2475-2489), a neutral gray tone was obtained, confirming that all color channels were properly balanced. We also employed one blank NOS slide as a '*reflectance standard strip*'. To verify the stable and consistent color appearance, we compared the recorded images and colors from the same NOS slide acquired in two different sessions. As shown in **Figure S5b**, the background images acquired in these two sessions, together with their corresponding colorimetric positions on the CIE chart (**Figure S5c)**, remained close to each other, with a measured color difference of ΔE_{00} = 0.51, well below the perceptual limit of human vision (JND= 2.3), confirming that the intrinsic stability of our illumination system ensures reliable cross-session color consistency.

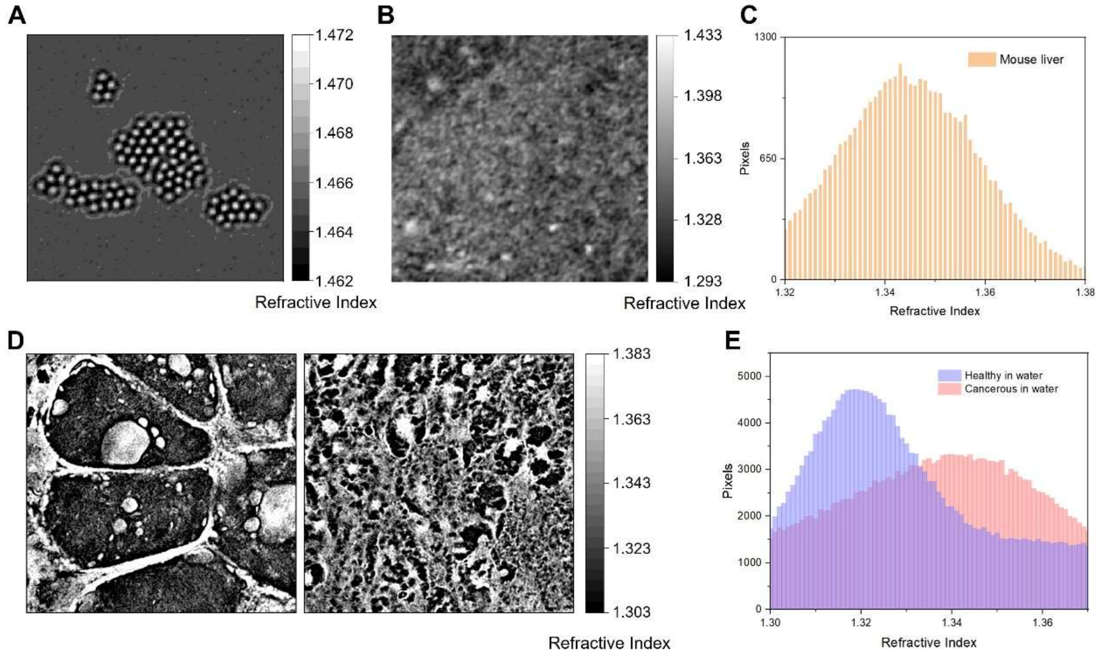

Figure S6. ODT calibration and validation on referenced samples. (a) Grayscale images of refractive index (RI) distribution maps of RI-matched beads (RI=1.462). (b) Grayscale images of RI distribution maps of fresh mouse liver tissue. (c) Statistical distribution of the RI values of tissue in (b). (d) Grayscale images of RI distribution maps of dehydrated healthy and cancerous colon tissue, soaked in water. (e) Statistical distribution of the RI values of two kinds of tissues in (d).

Note: We do recognize that most literature reported higher RI for tissues under fresh/hydrate state (e.g., *Optica* **2020**, *8(1)*, 6-14. RI: 1.35–1.38 for normal brain tissues; *Scientific reports* **2016**, *6(1)*, 1-10. RI: 1.33–1.35 for normal tissues). However, in our case, the tissue slices were observed under dehydrated state. As a dehydrate state tissue, voids/air gaps within and between tissue constituents is responsible for these smaller RI. To validate the accuracy of our measured RI using ODT technology, we performed extra supporting experiments.

(1) ODT calibration: To confirm that the ODT system was properly calibrated, we measured commercially available silica microspheres as a standard reference (nominal refractive index = 1.462 at 589 nm). Considering that our ODT laser operates at 532 nm, the expected refractive index of silica increases slightly due to material dispersion. As shown in **Figure S6a**, the reconstructed RI values were distributed around 1.463 at the periphery and 1.470 at the center, consistent with the expected range after accounting for system and dispersion effects.

(2) Fresh-tissue control: ODT measurement on freshly excised mouse liver (**Figure S6b**) produced RI histograms centered at 1.33–1.36 (mode ≈1.343) (**Figure S6c**), consistent with hydrated-tissue values reported in the literature (*Scientific reports* **2016**, *6(1)*, 1-10. RI: 1.33–1.35 for normal tissues). This confirms that our system is capable of producing physically consistent RI distributions when samples are prepared under conventional hydrated conditions.

(3) Medium-swap control: in this revision, we performed ODT measurements on dehydrated tissue sections immersed in water as the medium (**Figure S6d**). In this case, both healthy and cancerous regions exhibited peak positions (**Figure S6e**) that shifted toward the refractive index

of water (n = 1.33), further supporting the interpretation that the surrounding medium exerts a direct influence on the apparent RI distribution.

In summary, while we agree that the absolute RI values depend strongly on sample preparation processes, our calibration with extra samples confirms the accuracy of our system.

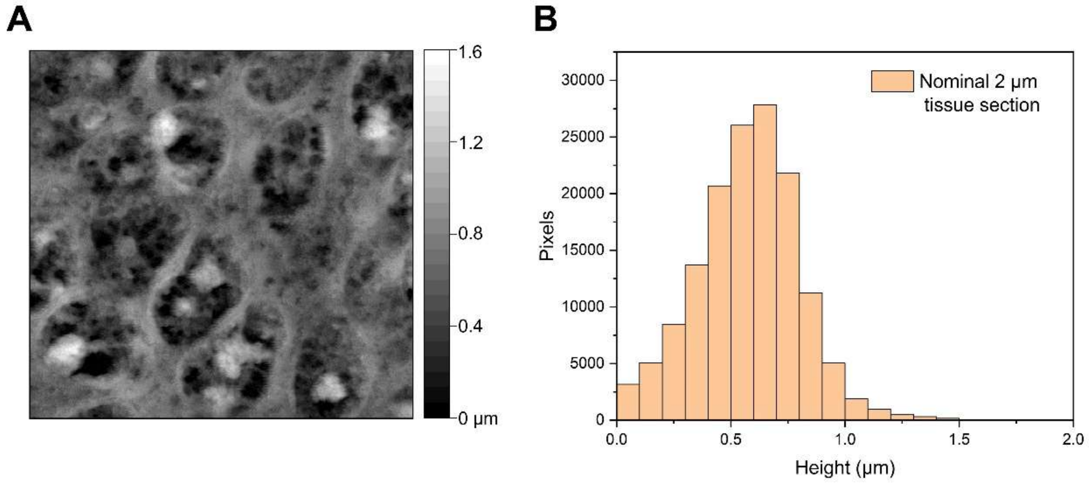

Figure S7. AFM characterization of nominal 2-µm tissue sections on NOS slides. (a) AFM-derived height-distribution map of a dewaxed nominal 2-µm section on the NOS slide. (b) Height-distribution histogram of the nominal 2-µm section in (a).

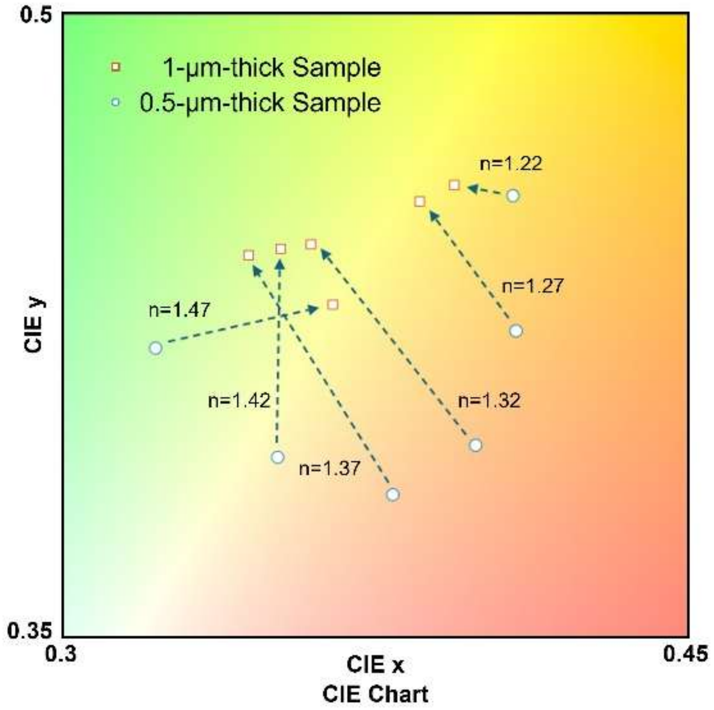

Figure S8. Simulated color position shifts on green NOS slides for 0.5 μm and 1 μm thick materials (n = 1.22-1.47) on the CIE chart. The dashed lines indicate the color shifts observed among samples of different thicknesses at a fixed RI.

Note: Our intension is to claim that this coupled effect of RI and thickness to the reflected color is the dominant mechanism responsible for the color difference observed from the NOS slide. In the main text, Figures 2c-d primarily discuss the color variations arising from differences in refractive index. However, by combining the original plots in **Figures 2c-d** into a single diagram, as shown in **Figure S8**, we traced the color trajectory as thickness increases from 0.5 to 1 μm, as indicated by dashed arrows. Distinct hue shifts can be observed, confirming that thickness also plays a key role in determining the color variation.

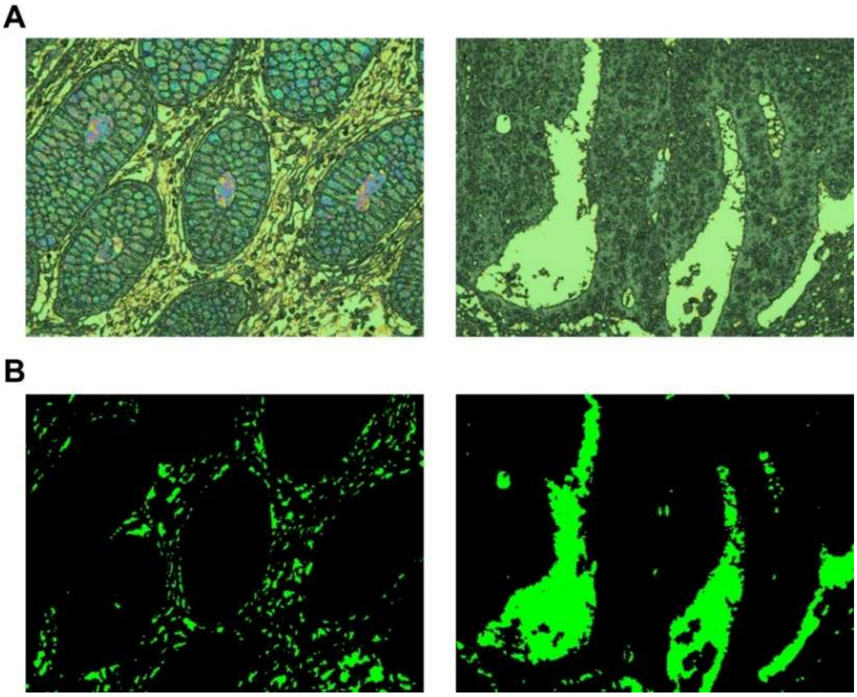

Figure S9. Evaluation of surface glare artifacts on NOS slides. (a) A representative healthy colorectal epithelial region and a representative colorectal adenocarcinoma epithelial region. (b) Results of brightness binarization analysis for the two images shown in (a).

Note: Possible artifacts in reflection mode would affect the quality of the images. Here we clarify, in principle, the three possible reflection-mode artifacts do not affect our system:
A. Surface glare: In this revision, we evaluated the possibility of specular (reflection) glare and veiling glare from surface by image analysis.

(1) Specular glare: According to the previous literature (*Sensors* **2023**, *23(2)*, 974.), a localized specular glare may occur on the tissue section surface due to mirror-like reflection from smooth or incompletely dewaxed regions, producing near-saturated, high-intensity highlights corresponding to areas of very high local reflectance that obscure underlying structural details. As the bare NOS slide only has a reflectance of ~40% (see **Fig. 1c** and **Fig. S1c**), any true glare artifact should be manifested as pixels in tissue regions with brightness higher than the NOS slide background.

To further reveal this possible artifact, in **Fig. S9**, we binarized images by intensity (i.e., from the original Fig. S9a to the binarized Fig. S9b), setting the threshold slightly above the brightness of the bare NOS slide background so that any pixels brighter than the slide itself stayed as green (positive) and others as black (negative). As a result, we did not observe such ‘positive’ pixels within tissue-covered regions, indicating that surface glare is not detectable under our imaging conditions.

(2) Veiling glare: Veiling glare on the tissue section, caused by diffuse scattering and internal reflections may reduce contrast and diminish overall image clarity. To verify whether our NOS-based reflection imaging exhibits any loss of visual sharpness compared with

conventional H&E transmission imaging, we evaluated nine no-reference sharpness metrics on matched regions from H&E and NOS slide in Fig. 2E in the main text. The tested metrics included Brenner gradient, Laplacian variance (vol), Modified Laplacian variance (vml), Tenengrad, spatial frequency (sf), LoG high-frequency energy (hfen), Fourier-domain high-frequency energy ratio (hf_energy_fft), entropy, and mean gradient(gmean_grad) (*Microscopy research and technique* **2004**, *65(3)*, 139-149; *Pattern Recognition* **2013**, *46(5)*, 1415-1432). As shown in **Table S4**, all nine indicators exhibited comparable values between the two modalities, with most ratios falling close to unity. These results confirm that, despite the presence of minor scattering-induced glare on the NOS slides, the reflection-mode images retain sharpness and contrast levels equivalent to those of conventional H&E transmission images, indicating that such artifacts are negligible and do not compromise the diagnostic interpretability of NOS-based histological imaging.

B. Mounting medium dependence: Our NOS slides are air-dried and examined without any coverslip or mounting medium (see details in Methods). Therefore, no mounting-medium-induced artifact is present.

C. Reduced depth averaging: According to the previous literature (*Physics in Medicine & Biology* **2013**, 58(11), R37.), reduced depth averaging, which physically arises from multiple scattering and the associated angular averaging, occurs only when the tissue thickness approaches or exceeds one mean free path ($1/\mu_s$, typically ~100 μm). In thick tissues, photons undergo repeated scattering events, during which their propagation angles are progressively randomized. This angular averaging leads to an exponential weighting of contributions from deeper layers, causing the measured reflectance to represent a depth-averaged mixture of subsurface structures rather than a sharply localized layer, which is the essence of the reduced depth averaging phenomenon. In contrast, when the tissue section is far thinner than one mean free path, as in our case (< 2 μm), light undergoes at most single or very few scattering events. Under such single-scattering conditions, angular averaging cannot occur, and therefore no depth-averaged reflectance is produced. Given that both specular glare (interface reflection) and veiling glare (systemic scattered light) have been experimentally excluded, it can be conclusively stated that no reflectance artifacts originating from reduced depth averaging are physically possible in our imaging configuration.

In summary, while we cannot perform the suggested comparative transmission/reflectance study, the above considerations support that the reflection-mode artifacts listed by the reviewer are negligible in our system.

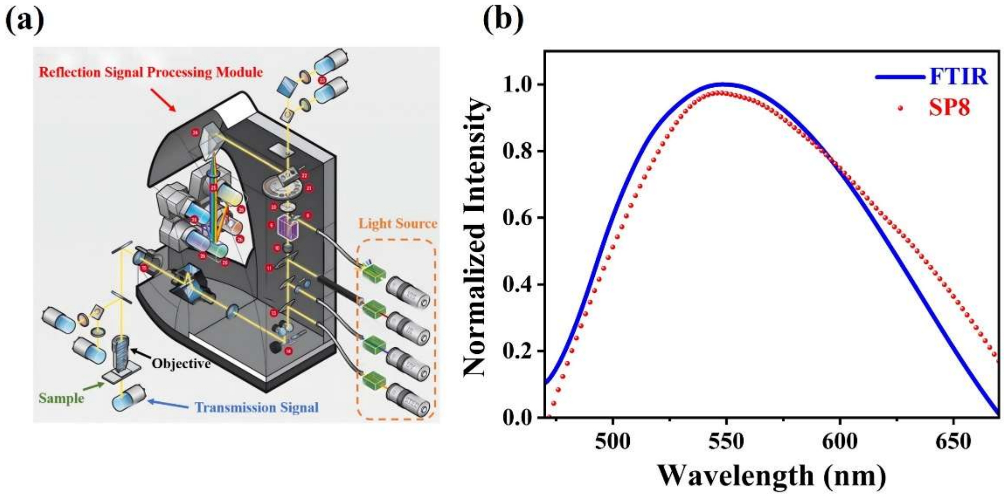

Figure S10. Epi-illumination configuration and spectral calibration framework of the Leica SP8 setup. (a) Configuration of Leica SP8. (b) Comparison of reflectance spectra measured from the same blank NOS slide region using the Leica SP8 (objective lens:4x and NA=0.13) and the HYPERION II FTIR microscope (objective lens:15x and NA=0.4).

Note: The system was mainly designed as a laser-scanning confocal microscope. However, under the epi-illumination configuration, the SP8 can function as a reliable microscopic spectroscopic module that enables wavelength-resolved reflectance measurements. The optical configuration of the system is shown in **Figure S10a**: both illumination and detection pathways are coaxial in this configuration. Broadband white-light is delivered through the objective lens onto the sample surface, and the back-reflected signal is collected through the same optical path. The reflected light is subsequently spectrally dispersed by a prism and detected by a hybrid photodetector array with high quantum efficiency in the visible range (470–670 nm). In our measurements, a 4× objective with a numerical aperture of 0.13 was used, providing a relatively low collection cone that approximates quasi-normal incidence. This geometry is directly comparable to the bright-field optical conditions typically used in pathology imaging, ensuring that the reflectance spectra are inherently linked to conventional bright-field micrographs.

To calibrate the system response and obtain quantitative reflectance spectra, we adopted a two-step workflow: (1) For each wavelength channel in the SP8 system, we first acquired an image of a silver mirror, followed by the specimen image, with both measurements performed under identical illumination and detection conditions. (2) The specimen signal was then normalized by the mirror reference to yield the calibrated reflectance spectrum.

This calibration strategy is widely employed in reflectance hyperspectral imaging, where spectral image stacks form a three-dimensional data cube (two spatial dimensions × wavelength) and reflectance is normalized against a reference standard (*Accounts of Chemical Research* **2016**, *49(10),* 2070-2079). Comparable procedures have also been validated in liquid crystal tunable filter (LCTF)-based hyperspectral imaging systems (*Optics Express* **2018**, *26(19)*, 25226-25243). This confirms that the adopted calibration workflow is a broadly applicable means of obtaining quantitative reflectance spectra, especially from microscopic images.

Next, we address how the spectral signals obtained from the SP8 system correspond to

bright-field imaging and conventional reflectance measurements. To establish this correspondence, we first acquired the reflected signal from a blank NOS slide (Figure S4b) using the SP8 system, with a silver mirror serving as a background reference. For comparison, reflectance spectra were also collected from the same region using a Bruker HYPERION II FTIR microscope (i.e., the same procedure described in Methods and **Fig. 1c**). As shown in **Figure S10b**, the spectra obtained from the two instruments exhibited consistent profiles and coinciding peak positions.

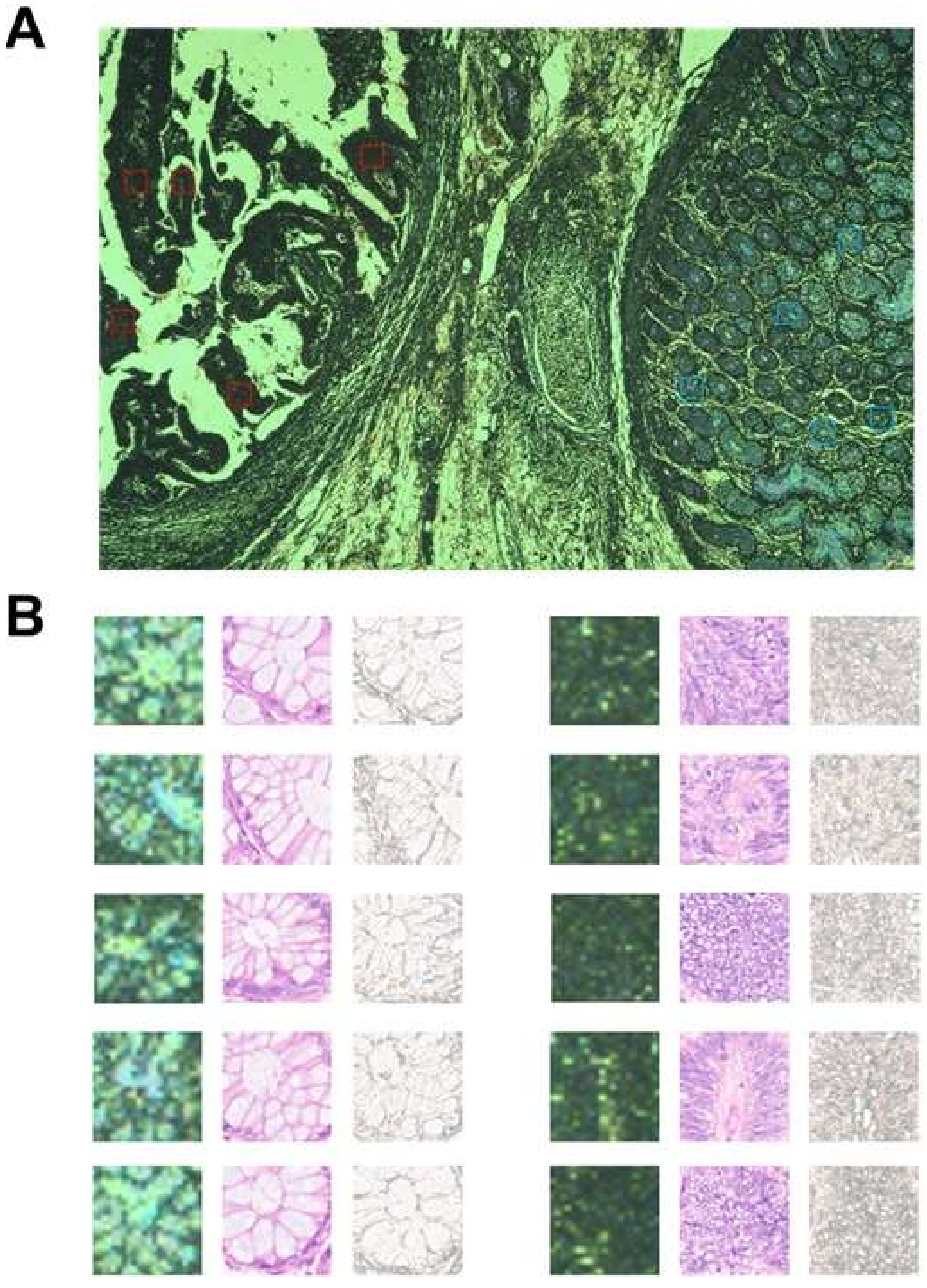

Figure S11. Selected tissue area on different slides for color comparison. (a) the selection of 5 healthy (and five cancerous areas from the same tissue image to ensure consistent imaging conditions between tissue types. (b) The corresponding selection of these ten areas across NOS, H&E, and non-stained sections.

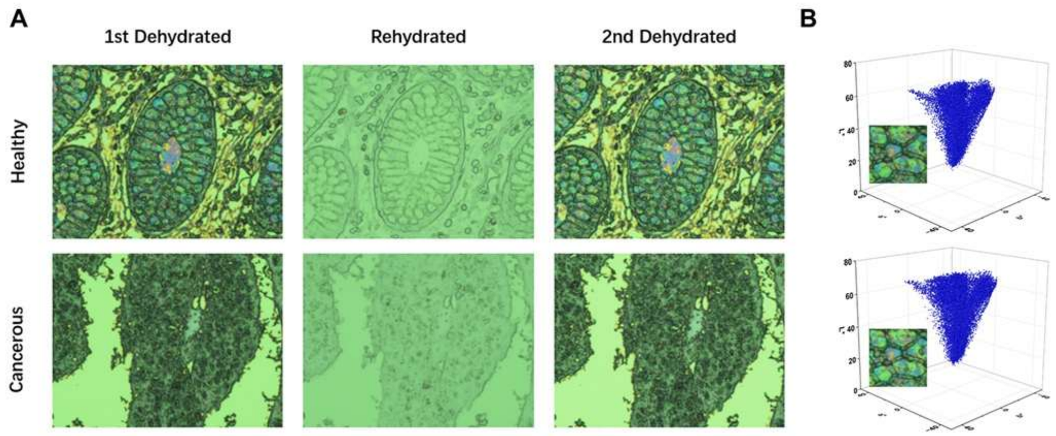

Figure S12. Validation of color stability under controlled rehydration/re-dehydration cycles. (a) Images of the same NOS region recorded after the first dehydration (left column), after rehydration (middle column), and after a second dehydration cycle (right column). Each column contains paired regions of healthy and cancerous colorectal epithelium. (b) Top: CIELAB point cloud from a selected region within normal colorectal epithelium after the first dehydration. Bottom: CIELAB point cloud from the corresponding region after the second dehydration, showing near-complete overlap.

Note: As stated in the Methods section, the exact end-state of tissue sections on NOS slides were air-dried without the use of mounting medium or cover glass. As the final step involved rinsing with double-distilled water, no solvent that could interfere with the refractive index of the air-dried sections should theoretically be present.

To directly address this, we performed the suggested control experiment by imaging the same NOS region under sequential dehydration/rehydration cycles. Specifically, we recorded the colorimetric response after the first dehydration, after rehydration, and after a second dehydration (**Figure S12a)**.

From the comparison between the first dehydration and the rehydration states, it is evident that the reintroduction of water substantially changes the optical appearance, confirming that hydration directly modulates the effective refractive index and, consequently, the observed structural color. However, when comparing the first and second dehydration states, the color response was nearly indistinguishable, with only marginal variations. In the corresponding CIELAB color space, the distributions of the two dehydration states show almost identical (**Figure S12b**), indicating that the colorimetric signature of NOS slides is highly reproducible once the samples are fully dried.

This control experiment demonstrates that under air-dried conditions, NOS slides consistently produce a stable and reproducible color output, confirming their strong resistance to environmental perturbations such as residual moisture and humidity fluctuations, with the optical response primarily determined by the intrinsic nanocavity configuration.

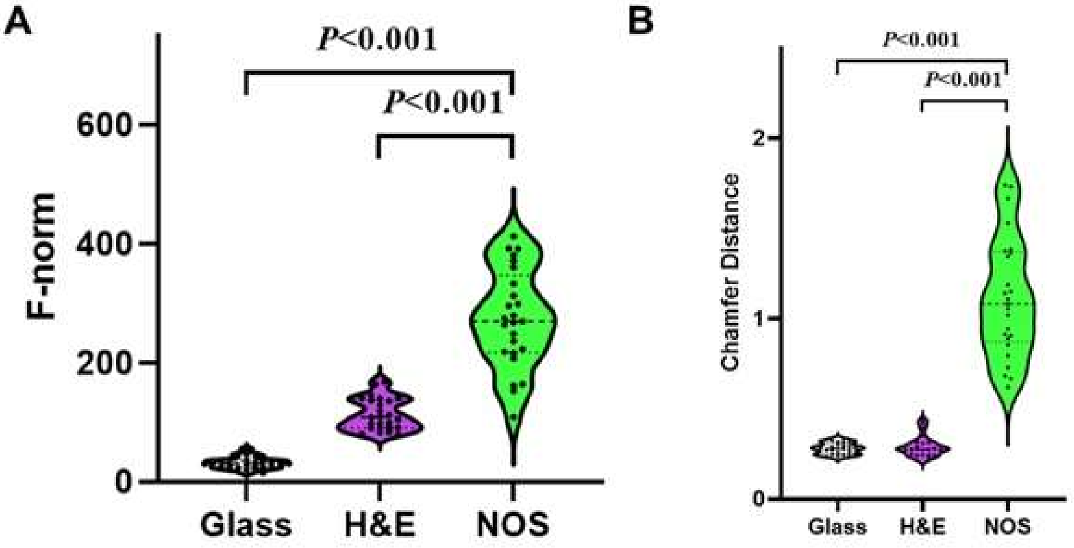

Figure S13. Frobenius norm (F-norm) and Chamfer Distance as metrics for evaluating differences between the color point clouds of healthy and cancerous tissues.

(a) Violin plots of F-norm of the cloud points between healthy/cancerous point clouds. A small Frobenius norm difference typically indicates that two three-dimensional point clouds are very similar in shape and size, whereas a large difference highlights significant disparities in shape or inconsistencies in dimensions between the point clouds. Two-sided t-tests were conducted to determine the statical significance of F-norm values of different slides. The *P* values are 3.07×10^{-19} for Glass vs NOS and 3.30×10^{-12} for H&E vs NOS, both below the threshold of $P<0.0001$.

(b)Violin plots of Chamfer Distance of the cloud points between healthy/cancerous point clouds. The calculated Chamfer Distance value quantifies the overall geometric discrepancy between the two 3D point clouds, where a lower value indicates a closer match, and a higher value highlights more pronounced structural differences. Two-sided t-tests were conducted to determine the statical significance of Chamfer distance values of different slides, and remarkably, the *P* values are 9.38×10^{-17} for Glass vs NOS and 2.30×10^{-16} for H&E vs NOS, both below the threshold of $P<0.0001$.

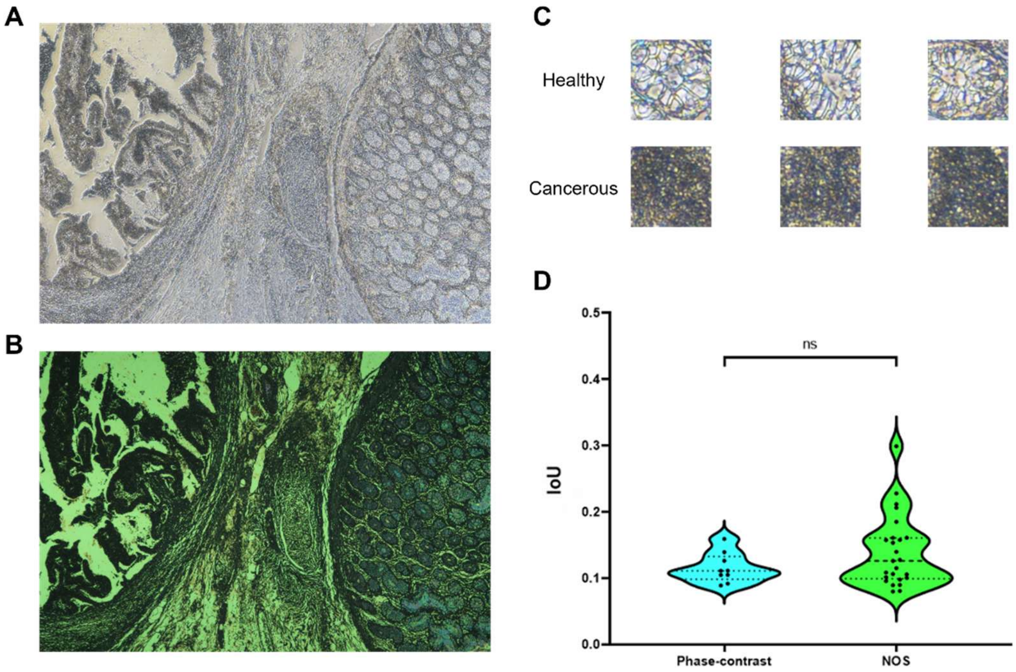

Figure R14. Comparison with phase-contrast. (a) Phase-contrast micrograph of the representative nonstained tissue region on a glass slide. (b) Brightfield micrograph of the representative nonstained tissue region on the green NOS slide, identical to **Figure S11a** in **Supporting Information**. (c)The corresponding selection of six areas (3 healthy and 3 cancerous) from (a). (d) Violin plots of the intersection over union (IoU) of cloud points between healthy/cancerous point clouds of samples on slides (Center dashed line indicates the median and the upper/lower dashed lines indicate the IQR; n = 9 for the phase-contrast group and n=25 for the NOS group). Using a two-sided Welch's independent-samples t-test, the p-value was 0.0766 for phase-contrast vs. NOS, which did not reach statistical significance at α=0.05.

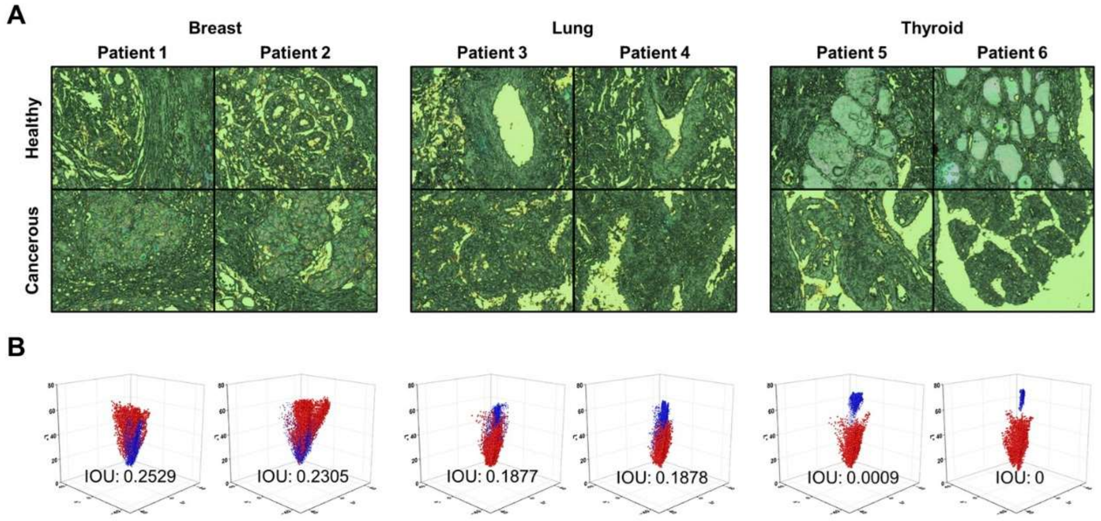

Figure S15. Colorimetric analysis across multiple tissue types on NOS slides. (a) Images of lung, breast, and thyroid tissue sections on NOS slides (n=2 cases per organ), each containing both healthy and cancerous regions. (b) CIELAB point-cloud distributions comparing healthy versus cancerous regions from the cases in (a).

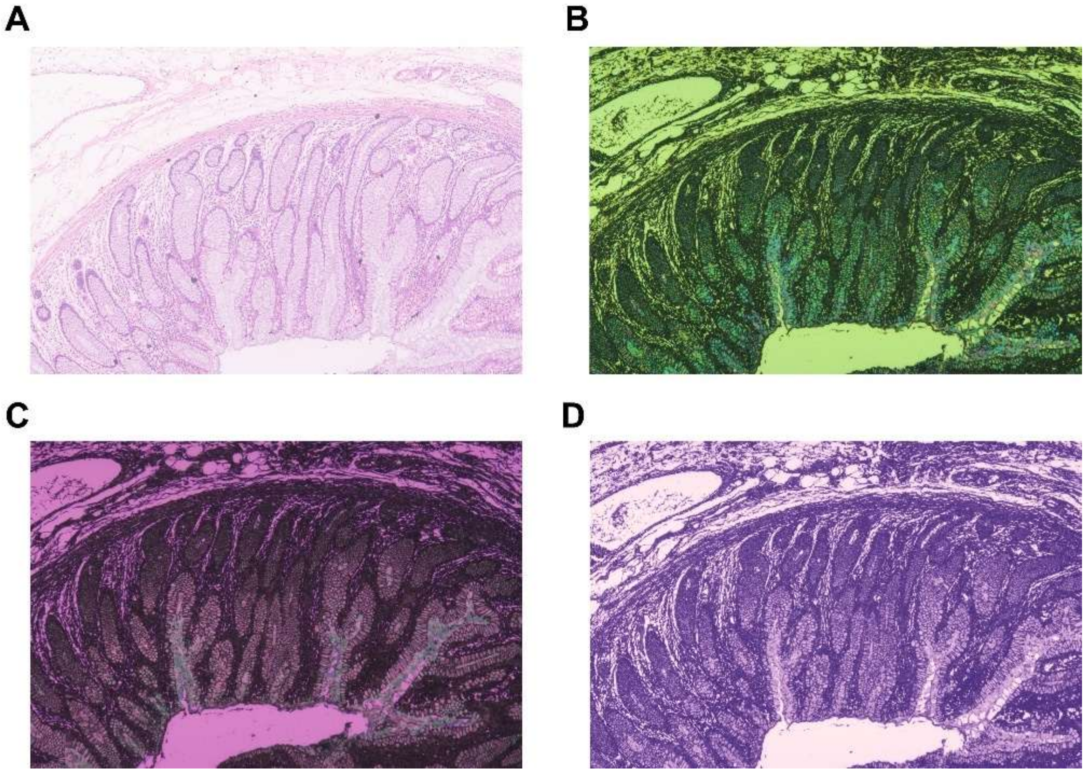

Figure S16. Comparison of conventional and NOS-based color schemes for histological visualization, showing (a) H&E-stained glass slide, (b) green NOS slide (nonstained), (c) purple NOS slide (nonstained), and (d) LUT-converted pseudo-H&E image from (b).

Note: The gold-standard color for regular pathology is the pink-purple color from H&E staining. However, the color of the image shown in the pioneering paper [*Nature* **2021**, *598(7879)*, 65-71] was in the yellow-green range. To directly compare this pioneering work, we developed an NOS slide in green in the current manuscript. We also developed pink colored NOS in Fig. 1b & Fig. 1c, and collected images in pink (as will be shown below). To further enhance interpretability and visual consistency with conventional pathology, additional experiments were conducted as follows:

(1) Color mapping on images from NOS slides: Following the color mapping procedure reported by (*Nature Communications* **2023**, *14(1)*, 1572), we transformed the original structural colors into a pseudo-H&E color scheme using a predefined look-up table (LUT), which emulates the conventional hematoxylin-eosin appearance for more familiar histological interpretation. Specifically, for each colorectal region, we collected four corresponding images: H&E-stained tissue on glass slides (**Figure S16a**), nonstained green NOS slides (**Figure S16b**), nonstained purple NOS slides (**Figure S16c**), and green NOS slide images converted to an H&E-like palette through LUT-based color mapping (**Figure S16d**). For the LUT-based transformation, the original NOS image was intensity-standardized by percentile normalization to suppress outlier brightness fluctuations, and its luminance distribution was subsequently remapped through a continuous lookup table designed to emulate the overall chromatic balance of hematoxylin–eosin staining. The mapping employed a purple-pink-shifted colormap, thereby yielding an appearance consistent with the visual characteristics of conventional H&E

coloration. Ten representative sets of these four corresponding images were prepared and independently reviewed by pathologists, covering all key histological layers of colorectal tissue were comprehensively evaluated, as illustrated in Fig. 3 of the main text.

(2) A reader study: As suggested by the reviewer, in this revision, a small reader study was conducted involving six practicing pathologists from Huashan Hospital. Each pathologist independently reviewed ten representative colorectal regions imaged under four color schemes mentioned above, without prior information on slide type.

Qualitative feedback (see **Table S8**) further indicated that the LUT-converted (i.e., pink-colored) NOS images yielded a clean visualization, with lighter backgrounds and stronger contrasts between nuclei and cytoplasm, thereby facilitating more confident visual interpretation. Meanwhile, the native green NOS slides were noted for their richer structural-color variations, particularly in mucinous and goblet cell regions, offering enhanced natural contrast and texture visibility. These additional studies demonstrated the flexibility and potential utility of the NOS platform for improving visual familiarity, motivating future rigorously designed multi-reader studies.

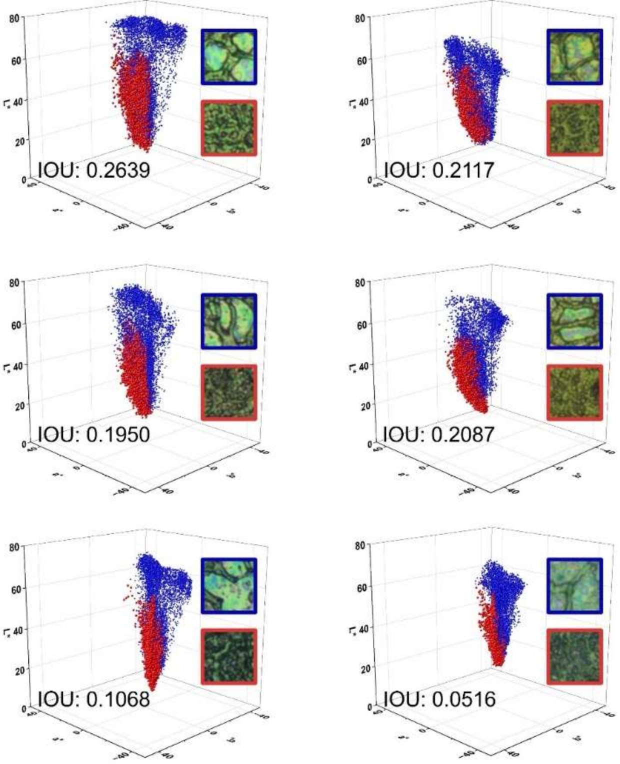

Figure S17. CIELAB color-space distributions comparing correctly classified healthy and cancerous epithelial regions from six pathologist-verified cases on NOS slides. Each scatter plot corresponds to one case, illustrating color clusters from both healthy (Blue) and cancerous (Red) tissue sections.

Note: Throughout this study, all "healthy vs. cancer" computations refer specifically to the comparison between normal colorectal epithelium and conventional colorectal adenocarcinoma. In real-world practice, misinterpretations on NOS slides can still occur. This is partly because colorectal disease often follows a continuum of progression rather than a strictly dichotomous state, and partly because non-conventional subtypes (e.g., mucinous adenocarcinoma) may exhibit structural-color and CIELAB characteristics that are closer to those of normal tissue, thereby reducing separability and increasing confusion. Therefore, in this proof-of-concept work, we intentionally focus our quantitative analysis on this common and well-defined binary setting, while treating gradual progression and subtype-related confounders as key limitations and important directions for future investigation. Within this defined scope, the IoU differences we report are indeed robust and consistently observed across the evaluated samples. Larger and more diverse cohorts spanning grades and subtypes will be required to systematically evaluate these effects and to extend the analysis/modeling strategies for robust NOS interpretation under clinically heterogeneous conditions.

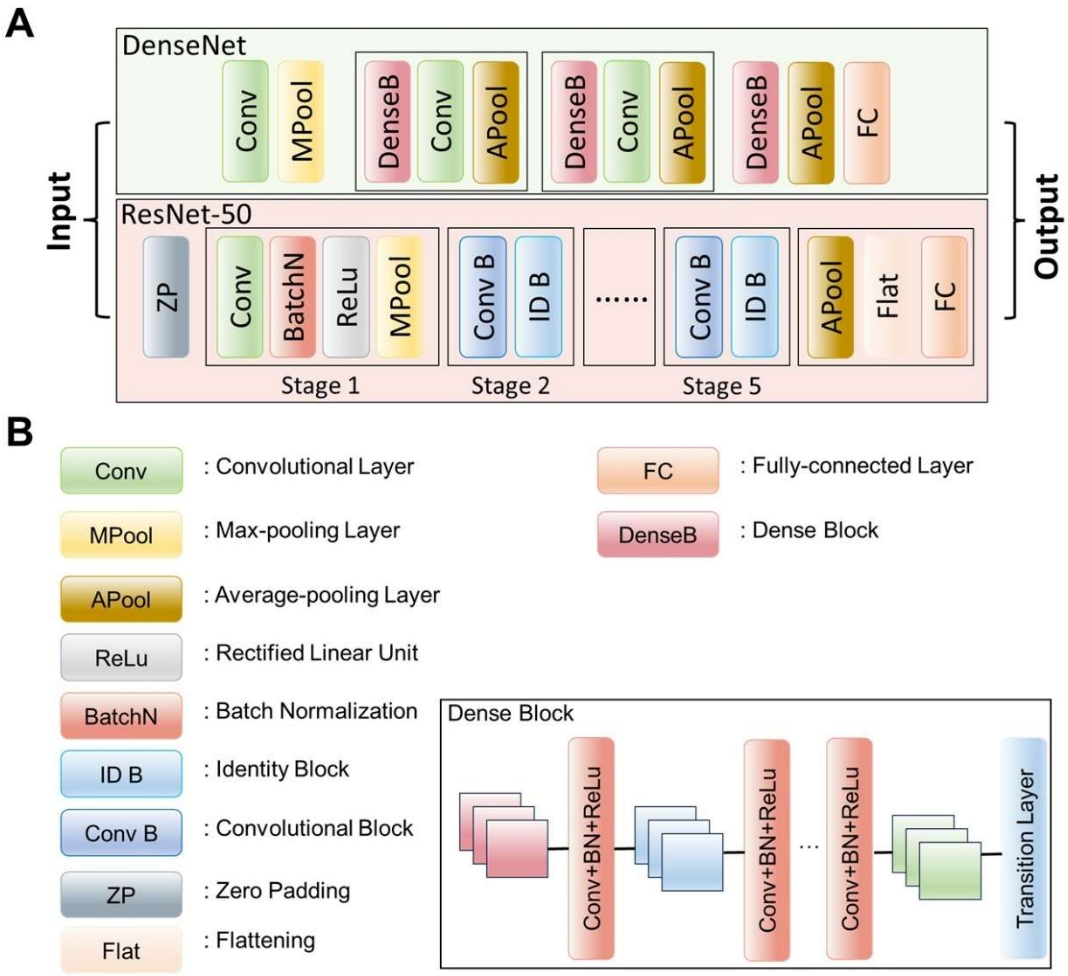

Figure S18. Structure of AI classification model. (a) Schematic illustration of the implementation and comparison of two convolutional neural network architectures: DenseNet121 and ResNet50. (b) Schematic illustration of a detailed explanation of the terminology used. All experiments were conducted using a single NVIDIA Tesla A100 GPU with 80 GB of HBM2 memory. We evaluated two convolutional neural network backbones—DenseNet-121 and ResNet-50—each adapted for binary classification by replacing the original fully connected layer with a two-class linear output. The DenseNet-121 model includes approximately 8.0 million trainable parameters, while ResNet-50 contains around 25.6 million. Input images were resized to 224 × 224 pixels, and training was performed in full-precision (FP32) with a batch size of 32. Under these settings, DenseNet-121 required approximately 8 GB of GPU memory, whereas ResNet-50 consumed about 12 GB to support both forward and backward passes.

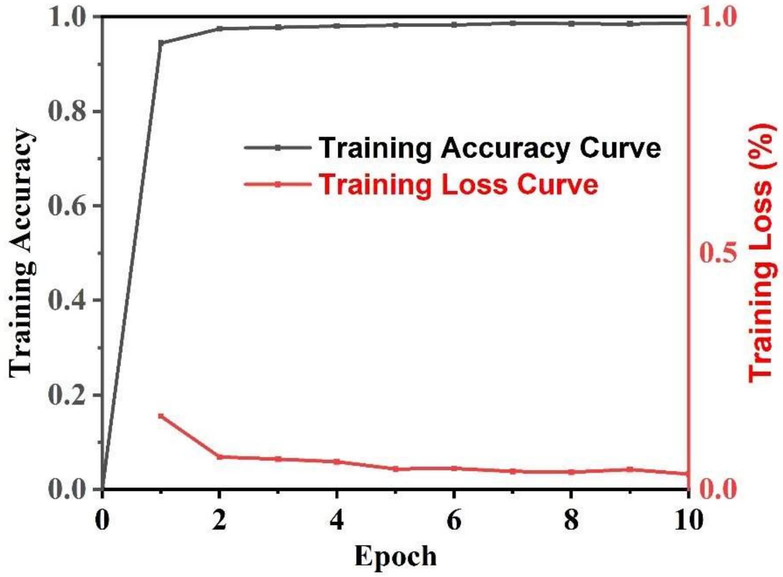

Figure S19. Trends of Training loss and accuracy over epochs for DenseNet-121. Training loss decreases rapidly with a steady increase in training accuracy, indicating effective learning.

A

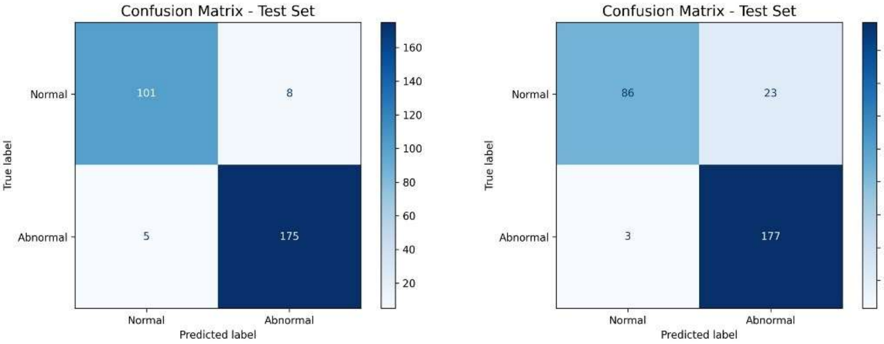

B

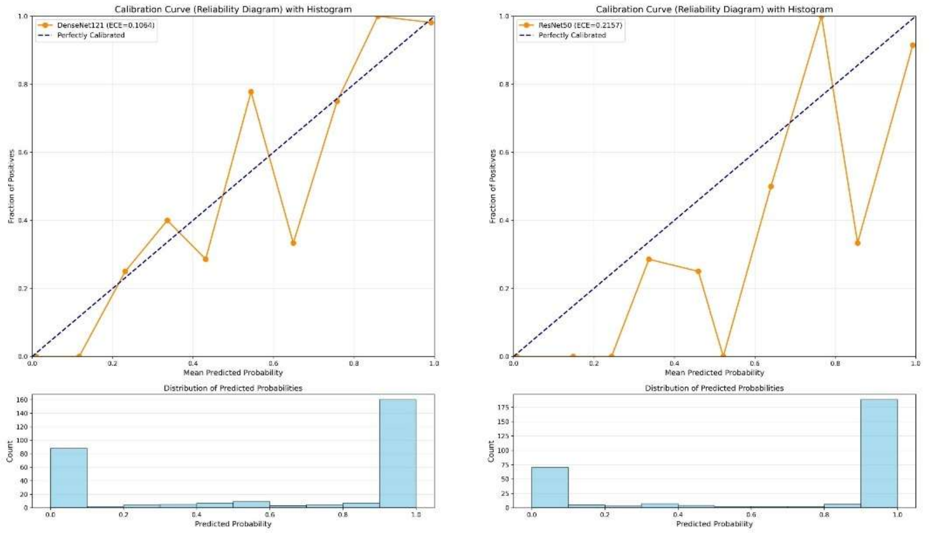

C

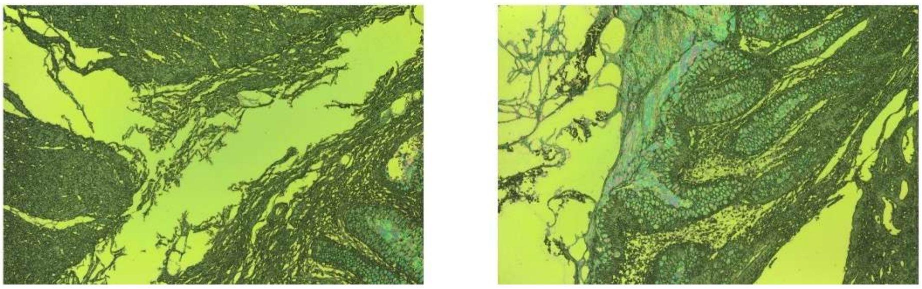

Figure S20. Summary of model evaluation results, illustrating classification accuracy, probability calibration, and representative failure cases. (a) Confusion matrices from the two trained models based on the revised patient-wise splits. (b) Calibration curves and probability histograms for both models. (c) Representative failure exemplars, where the left panel shows the model-identified false positive case and the right panel shows the false negative case.

Note: To comprehensively assess the diagnostic reliability of the trained models, we analyzed their confusion patterns, probability calibration, and representative failure cases.

(1) Confusion patterns: The confusion matrices, as shown in **Figure S20a**, reveal exceptional classification performance for both architectures on the colon cancer detection task. DenseNet demonstrates outstanding discriminative capability with 175 true positives and 101 true negatives, yielding an overall accuracy of 95.5%. The model exhibits remarkably low error rates with only 5 false negatives (2.8% of actual abnormal cases) and 8 false positives (7.3% of actual normal cases). ResNet shows comparable but slightly different performance characteristics, achieving 177 true positives and 86 true negatives with an overall accuracy of 91.0%. While ResNet50 demonstrates marginally higher sensitivity (98.3%) with only 3 false negatives, it exhibits significantly more false positives (23 cases, representing 21.1% of actual normal cases). This trade-off reflects the model's bias toward predicting abnormal cases.

(2) Calibration analysis: The calibration curves, as shown in **Figure S20b**, provide critical insights into the reliability of predicted probabilities. DenseNet exhibits an Expected Calibration Error (ECE) of 0.1064, representing substantially improved calibration compared to ResNet50's ECE of 0.2157. The lower ECE indicates that DenseNet's probability estimates are more trustworthy for clinical decision-making.

DenseNet's calibration curve demonstrates relatively good alignment with the perfect calibration diagonal across most probability ranges. Notable calibration characteristics include reasonable performance in the low-to-mid probability range (0.0-0.5), though some fluctuation exists around 0.3-0.4 and 0.5-0.6 ranges. Critically, the model shows strong calibration in the high-confidence region (0.8-1.0), where the curve closely follows the ideal diagonal. The probability distribution histogram reveals a pronounced bimodal pattern with the majority of predictions concentrated at the extremes (approximately 87 cases near 0.0 and 157 cases near 1.0), indicating that the model makes highly confident predictions for most samples, with relatively few ambiguous cases.

ResNet50's calibration curve shows substantial deviations from perfect calibration, particularly in the mid-range probabilities (0.3-0.6). The most concerning issue is the severe miscalibration around 0.5, where predicted probabilities near 0.5 correspond to actual positive fractions dropping to approximately 0. This suggests the model exhibits poor discrimination in uncertain cases, making unreliable probability estimates when it should be expressing uncertainty. The high-confidence region (>0.9) shows better calibration, though a notable dip occurs around 0.9 where the fraction of positives drops to approximately 0.33 despite high predicted confidence. The probability distribution is similarly bimodal (approximately 70 cases near 0.0 and 182 cases near 1.0), indicating strong conviction in most predictions but with questionable reliability in the intermediate range.

(3) Failure exemplars: By examining the model’s misclassified results, we selected one representative tile from the false positive cases and one from the false negative cases as failure exemplars for detailed analysis, as shown in **Figure S20c**. For example, in the false positive case (left panel), the image contained a small portion of healthy epithelial tissue together with a large area of muscular tissue. Because the training set did not include a separate category for non-epithelial tissue (as the classification was binary: healthy vs. cancerous), the model may have misinterpreted the muscle texture or color features as abnormal tissue. Similarly, in the

false negative case (right panel), the image actually contained both healthy and cancerous tissue regions. Since our training dataset did not include such "mixed-type" tiles, the model may have been influenced by the dominant features of the healthy region and consequently classified the tile as normal, overlooking the smaller cancerous area. From these failure exemplars, we learned that the model tends to misclassify tiles containing mixed or underrepresented tissue types. In future work, we will expand the training dataset to include a broader range of tissue categories and mixed regions and explore multi-class or hierarchical classification strategies to further improve diagnostic accuracy and generalization.

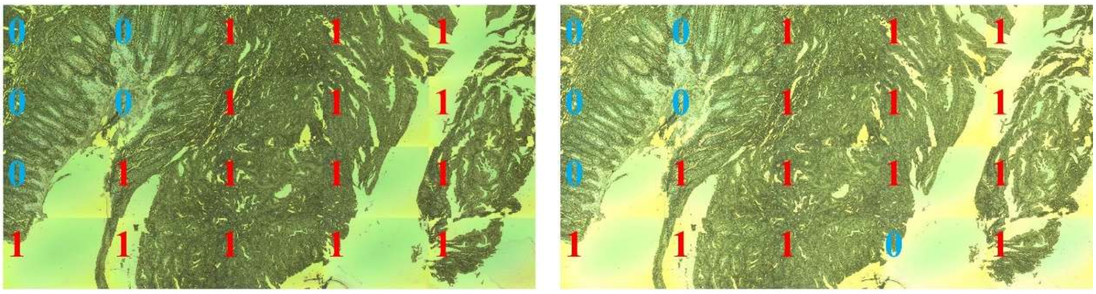

Figure S21. Comparison of model classification results using the dataset under different optical conditions. The two panels show the predictions obtained from the original dataset (left) and the brightness-adjusted dataset (right), both evaluated using the same trained model. In the prediction maps, ‘0’ denotes regions classified by the model as normal, and ‘1’ denotes regions classified as abnormal.

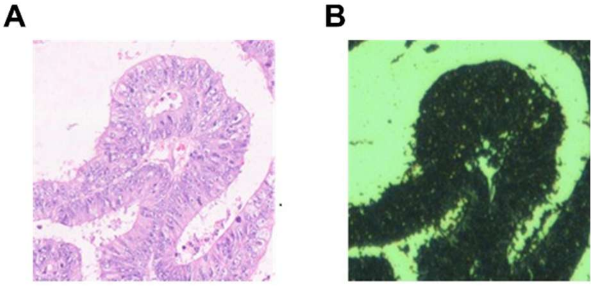

Figure S22. Representative image regions of serial sections, including (a) H&E-stained & (b) NOS nonstained, selected from **Fig. 2e** for quantitative evaluation of registration errors.

Note: The 'registration error' is the geometric misalignment between corresponding lesion regions in the H&E ground-truth slide and the adjacent NOS section after image registration. To quantitatively assess this discrepancy, we computed the average symmetric surface distance (ASSD), a standard metric for evaluating boundary-level registration accuracy in medical imaging [*Medical Image Analysis* **2009**, *13(5)*, 605–619]. This metric provides an intuitive, interpretable measure of geometric registration quality and is particularly suited for serial-section alignment, where small local distortions or tissue losses are expected during microtomy.

(I) The calculation of ASSD is divided into the following steps:

1. **Inputs:** Let A denote the pathologist-annotated lesion mask on the H&E section (**Figure S22a**) and B the lesion mask on the NOS section after registration (**Figure S22b**) (both binary, same field-of-view). The pixel pitch is s µm/pixel (here s=1.58 µm/pixel), so all distances can be reported in μm.
2. **Pre-processing and common grid:** We resample A and B to a common raster (same spacing ss and image size). If the registration involves sub-pixel warps, we re-quantize via a signed-distance interpolation to avoid jagged edges. To suppress tiny speckles, we remove connected components smaller than a few pixels (area filter).
3. **Boundary extraction:** We compute perimeter sets ∂A and ∂B using a morphological perimeter operator (equivalently, a morphological gradient on the binary masks). Each boundary set is a list of pixel coordinates $\{a_i\}_{i=1}^{N_A}$ and $\{b_i\}_{j=1}^{N_B}$. We then convert pixel coordinates to physical coordinates by multiplying by s.
4. **Nearest-boundary distances (both directions):** For every $a_i \in \partial A$, we compute its shortest Euclidean distance to ∂B:

$$d(a_i, \partial B) = \min_{b_j \in \partial B} \left\| a_i - b_j \right\|_2$$

Likewise, for every $b_j \in \partial B$,

$$d(b_j, \partial A) = \min_{a_i \in \partial A} \left\| b_j - a_i \right\|_2$$

To make this efficient and numerically stable, we use distance transforms: build a binary image of ∂B, compute its Euclidean distance map $D_B(\cdot)$, and evaluate D_B at pixels of ∂A (and vice versa using D_A at ∂B). This avoids $O(N_A N_B)$ pairwise searches.

5. **ASSD definition and reporting:** The Average Symmetric Surface Distance is then

$$ASSD(A,B)=\frac{1}{|\partial A|+|\partial B|}\Big(\sum_{a\in\partial A}\min_{b\in\partial B}d(a,b)+\sum_{b\in\partial B}\min_{a\in\partial A}d(b,a)\Big)$$

reported in μm (distances in pixels multiplied by s).

By applying the procedures described in Steps 1-5 above, we obtained the ASSD between the H&E and NOS lesion boundaries. The computed ASSD was 8.3 μm, confirming that the geometric alignment between adjacent serial sections is highly precise, and that the residual registration error lies well within the normal biological variability observed between consecutive tissue slices.

(II) Evaluation of the sensitivity of κ to boundary offsets.

To address the reviewer's suggestion to perform a sensitivity analysis by dilating or eroding the lesion masks (±25–50 μm) in order to bound κ, we conducted a detailed evaluation of Cohen's κ and its dependence on boundary perturbations. First, we introduce the computation workflow for κ. Using the two aligned binary masks obtained from the H&E annotation (**Figure S22a**) and the registered NOS section (**Figure S22b**), we computed the pixel-wise confusion counts: TP=#(A=1,B=1),TN=#(A=0,B=0), FP=#(A=0,B=1), and FN=#(A=1,B=0), where N is the total number of pixels within the evaluation region. The observed agreement is p_o=(TP+TN)/N; the marginal positive rates are p_A=(TP+FN)/N and p_B=(TP+FP)/N; and the chance agreement is $p_e=p_Ap_B+(1-p_A)(1-p_B)$. The Cohen's κ statistic is then calculated as: $\kappa=(p_o-p_e)/(1-p_e)$. For the baseline comparison between the registered pair (same ROI), this yields κ=0.549.

Then, following the above procedure, we evaluated the sensitivity of κ to boundary offsets. Because κ equally weights all pixels, it is inherently sensitive to small boundary displacements. To quantify this sensitivity, we applied controlled morphological dilations and erosions to one of the masks, introducing synthetic boundary shifts of ±Δ μm while keeping the lesion interior unchanged. The structuring element radius was r=Δ/s pixels, where s is the pixel size (1.58 μm/pixel). κ was recalculated for each Δ to assess how geometric perturbations influence agreement. The results show strong dependence on boundary offsets: κ=0.228 after a ±25 μm erosion, and approximately 0.03 under ±25–50 μm dilations or erosions. This pronounced variation is expected because κ penalizes every pixel equally regardless of spatial relevance; thus, even minimal shifts along lesion boundaries can dramatically affect the numerical value of κ without representing a meaningful loss of alignment. Importantly, this behavior does not undermine our conclusions. First, the measured registration error (ASSD = 8.3 μm) is substantially smaller than the perturbations that cause κ to decline sharply, confirming that true geometric mismatch is minor. Second, lesion interiors remain well aligned, as verified visually in **Figure S22** and by the consistent glandular morphology across sections. Third, in this study, the H&E annotations are used as morphological ground truth for comparison with NOS-based classification rather than for pixel-by-pixel label matching. The demonstrated cellular-scale correspondence therefore supports the validity of using adjacent serial sections for NOS training and evaluation.

Thickness	SiH_4 / NH_3/ N_2 (sccm)	Calibrated Rate	Processing Time
269 nm Si_xN_γ	23:20:980	~ 18 nm/min	15 min
Pressure 850 mTorr		RF Power 20.0 W	
	Unit price	Amount	Cost
Silicon Wafer	\$12/wafer	1/6 wafer	\$2
Si_xN_γ Deposition	\$3/wafer	1/6 wafer	\$0.5
Laser Cutting	\$2/wafer	1/6 wafer	\$0.33
Cleaning	\$1/wafer	1/6 wafer	\$0.17
Total Cost of one NOS slide			\$3

Table S1. PECVD deposition settings used to obtain the 269 nm Si_xN_γ layer, and laboratory-scale cost breakdown for fabricating one 25 × 75 mm NOS slide.

Note: Our NOS slides are fabricated through a simple process that involves depositing a single dielectric layer on silicon, followed by wafer-level cutting into slide formats (details in Methods). This process does not require photolithography, pattern transfer, or the use of precious metals, resulting in straightforward and low-cost fabrication. In our current lab workflow, six 25 × 75 mm slides are diced from one 6-inch Si wafer (Fig. 1a). The itemized cost per slide is ≈ \$3: silicon wafer \$2, Si_xN_γ deposition \$0.5, laser dicing \$0.33, and cleaning \$0.17. On the user side, NOS slides plug directly into routine histology: sections are mounted and dewaxed as usual, and imaging is performed on a standard bright-field microscope without added optics, polarizers, or staining steps. Compared with historical EBL/FIB-based plasmonic slides, this unpatterned NOS stack greatly simplifies both manufacturing and adoption in pathology labs.

Importantly, our cost comparison is framed against earlier plasmonic pathology slides fabricated by serial EBL/FIB at prototype scale. And we acknowledge that modern high-throughput nanolithography (e.g., Displacement Talbot Lithography) can achieve comparable per-unit costs for wafer-scale periodic nanostructures. Nevertheless, NOS remains advantageous for rapid, maskless iteration (e.g., thickness/material tuning without mask redesign) while preserving plug-and-play compatibility with routine histology workflows.

Moreover, for the outlook to industrial scale, at scale, per-slide cost can be further reduced via batch LPCVD/PECVD on 200-300 mm wafers or panel coaters, automated dicing/scribing, and inline cleaning/QA. These mature, high-throughput processes (already ubiquitous in flat-panel and semiconductor lines) are expected to drive costs well below current lab-scale values, while preserving full compatibility with existing histology workflows.

Dewaxing process	H&E staining process
Baked in an 80 °C oven for 20 min to flatten the sections and improve adhesion to the substrate.<br>Soaking in xylene for 5 minutes, 3 times<br>Soaking in EtOH for 2 minutes, 3 times<br>Soaking in water for 5 minutes.<br>Placing at 50% relative humidity for 30 min to air-dry the sections until no visible liquid residue remained.	Soaking in hematoxylin stain for 10 minutes<br>Soaking in water for 5 minutes<br>Soak in 0.5% HCl-alcohol solution for 30 seconds<br>Soaking in water for 5 minutes<br>Soaking in Eosin solution for 3 minutes<br>Soaking in EtOH for 1 minute, 3 times<br>Soaking in xylene for 2 minutes, 3 times<br>Mounting with neutral resin

Table S2. Dewaxing and H&E staining process. Non-stained sections on glass slides and NOS slides are required only to do dewaxing, while H&E-stained sections undergo both dewaxing and H&E staining.

Δh_{min} for perceptible color difference				Δn_{min} for perceptible color difference			
h (nm)	n	Δh_{min} (nm)	$\Delta h_{min}/h$ (%)	h (nm)	n	Δn_{min}	$\Delta n_{min}/n$ (%)
100	1.33	3.2	3.20	500	1.22	0.0179	1.47
300		4.5	1.50		1.27	0.0160	1.26
500		8.7	1.74		1.33	0.0273	2.05
700		17.5	2.50		1.37	0.0132	0.96
900		17.6	1.96		1.42	0.0098	0.70

Table S3. Quantitative evaluation of the minimal variations in tissue thickness (h) and refractive index (n) required to produce a perceptible color difference ($\Delta E_{00} = 2.3$, JND threshold) based on TMM simulations.

Note: To further evaluate the sensitivity of the reflected color to RI and thickness, we conducted an additional quantitative analysis. Specifically, we extended our previous TMM-based simulations to model the color responses of the on-chip structure while defining a perceptual color-difference threshold of $\Delta E_{00} = 2.3$, corresponding to the just noticeable difference (JND) perceived by the human eye (*Proceedings of the National Academy of Sciences* **2020**, *117(48)*, 30107-30117.). By incrementally varying either the RI or the thickness under different controlled conditions, we determined the smallest parameter change required to exceed this JND threshold. The results, summarized in **Table S3**, revealed that both the RI and thickness exhibit perceptual sensitivity to variations as small as 1–3%. These findings collectively confirmed that both parameters play crucial and comparable roles in governing the structural color variation of the tissue sections on NOS slide.

	H&E	NOS	Ratio (NOS/H&E)
Brenner	7518.94	8180.62	1.088
Vol	0.0440906	0.0567017	1.286
Vml	0.0356219	0.0596594	1.675
tenengrad	0.0284567	0.0288712	1.015
Sf	0.105385	0.113863	1.080
Hfen	0.000640819	0.000681853	1.064
hf_energy_fft	0.0178909	0.0204891	1.145
Entropy	2.64897	2.05623	0.776
gmean_grad	0.0541234	0.0476177	0.880

Table S4. Nine indicators with comparison of two images from different methods in **Figure S22**. (Details in **Figure S9**)

NOS	Healthy 1	Healthy 2	Healthy 3	Healthy 4	Healthy 5
Cancerous 1	0.095966971	0.108676806	0.206358217	0.13997724	0.100774704
Cancerous 2	0.105946374	0.115443875	0.228103492	0.157951504	0.106489825
Cancerous 3	0.080979442	0.089553617	0.161065252	0.126163074	0.080118778
Cancerous 4	0.153611503	0.160851915	0.299118482	0.2118492	0.157652177
Cancerous 5	0.090138824	0.101805604	0.185241081	0.127780933	0.098163419

H&E	Healthy 1	Healthy 2	Healthy 3	Healthy 4	Healthy 5
Cancerous 1	0.175942777	0.204674587	0.239394796	0.205034754	0.230597185
Cancerous 2	0.217011885	0.255537909	0.248911335	0.234676738	0.264402345
Cancerous 3	0.244410331	0.296224079	0.279995473	0.24377135	0.267896676
Cancerous 4	0.203436527	0.224637245	0.248993538	0.233862455	0.252997808
Cancerous 5	0.218850234	0.259769127	0.263827225	0.242753069	0.263474907

Non	Healthy 1	Healthy 2	Healthy 3	Healthy 4	Healthy 5
Cancerous 1	0.2402459	0.357322419	0.289199785	0.286562189	0.286989741
Cancerous 2	0.251871571	0.362595346	0.289940943	0.290812961	0.246305304
Cancerous 3	0.37051746	0.455300704	0.33826881	0.372983281	0.283220108
Cancerous 4	0.446436245	0.51282893	0.402090499	0.448804025	0.315002082
Cancerous 5	0.377469458	0.469297077	0.33933482	0.398894289	0.293548579

Table S5. IoU values of color points clouds between healthy and cancerous images under different methods.

NOS	Healthy 1	Healthy 2	Healthy 3	Healthy 4	Healthy 5
Cancerous 1	360.1772538	267.988304	263.9503315	207.4749073	380.645774
Cancerous 2	371.076263	278.8292047	274.6773094	217.8408568	391.6373621
Cancerous 3	392.1444542	298.8918207	295.8850171	236.828812	412.5378469
Cancerous 4	248.5973336	161.4861986	152.6238141	108.7493244	269.7596579
Cancerous 5	312.5612465	222.5401583	216.2100128	164.454814	333.4001516

H&E	Healthy 1	Healthy 2	Healthy 3	Healthy 4	Healthy 5
Cancerous 1	139.8147256	135.8149953	108.2156281	91.24620735	97.29817479
Cancerous 2	145.088161	140.9485355	111.7735793	90.41030853	99.26679178
Cancerous 3	83.4583661	80.56437171	82.61163645	119.1025884	98.25804699
Cancerous 4	140.610802	136.3736689	104.2961291	79.59089189	89.67500794
Cancerous 5	168.0948808	163.9064772	129.2862098	91.08781975	109.80425

Glass	Healthy 1	Healthy 2	Healthy 3	Healthy 4	Healthy 5
Cancerous 1	46.2435	27.99153339	33.96089001	30.33247387	25.4760252
Cancerous 2	55.73230713	35.40454023	36.14311562	33.57402904	24.72216111
Cancerous 3	24.21480813	19.69910879	35.29271901	31.35695981	37.39571483
Cancerous 4	43.11891762	21.82565313	22.44747048	19.97447469	14.82787996
Cancerous 5	46.37955494	28.18850724	34.16906022	30.61228422	25.94125201

Table S6. F-norm values of color points clouds between healthy and cancerous images under different methods.

NOS	Healthy 1	Healthy 2	Healthy 3	Healthy 4	Healthy 5
Cancerous 1	0.9158	1.7428	0.6694	1.3469	1.0549
Cancerous 2	0.8914	1.1888	0.6207	1.1429	0.8575
Cancerous 3	0.9073	1.668	0.6842	1.3661	0.9442
Cancerous 4	1.0818	1.3849	0.7304	1.3761	1.023
Cancerous 5	1.1502	1.7332	0.7956	1.5304	1.1081

H&E	Healthy 1	Healthy 2	Healthy 3	Healthy 4	Healthy 5
Cancerous 1	0.2871	0.2824	0.244	0.241	0.2766
Cancerous 2	0.2508	0.2445	0.2271	0.2142	0.2409
Cancerous 3	0.3074	0.2865	0.2748	0.2762	0.2758
Cancerous 4	0.4396	0.4212	0.3516	0.2918	0.3946
Cancerous 5	0.3198	0.3176	0.272	0.2741	0.3136

Glass	Healthy 1	Healthy 2	Healthy 3	Healthy 4	Healthy 5
Cancerous 1	0.3179	0.2804	0.2944	0.2728	0.2847
Cancerous 2	0.2562	0.2356	0.2464	0.2382	0.2343
Cancerous 3	0.2799	0.2487	0.2667	0.2509	0.2505
Cancerous 4	0.3174	0.287	0.2955	0.2717	0.2887
Cancerous 5	0.3305	0.3029	0.324	0.3381	0.3173

Table S7. Chamfer Distance of color points clouds between healthy and cancerous images under different methods.

Diagnostic criterion	Consensus summary among six pathologists
Goblet cells & mucin pools visibility	All six pathologists agreed that goblet cells and mucinous components were clearly identifiable in all NOS color schemes, comparable to H&E. Green NOS slightly enhanced mucin pools contrast by exhibiting richer color variations.
Nuclear morphology	All reviewers confirmed clear nuclear morphology across NOS and H&E images.
Epithelial–stromal distinction	Consensus agreement that epithelial and stromal layers were well delineated under all NOS modes.
Smooth muscle orientation	All reviewers reported that smooth muscle fibers in the muscularis propria were clearly distinguishable.

Table S8. Summary of qualitative evaluations from six practicing pathologists in the reader study. (Details in Figure S16)

	Healthy, H&E	Cancerous, H&E	Non-epithelial, H&E	Row Total
Healthy, NOS	752	0	0	752
Cancerous, NOS	11	3342	24	3377
Non-epithelial, NOS	0	27	1991	2018
Column Total	763	3369	2015	6147

Table S9. Results of Statistical analysis of pathological diagnoses on NOS slides, compared with H&E staining. From the results, the Cohen's κ can be calculated as follows:

$$P_0 = \frac{752 + 3342 + 1991}{6147} = 0.990$$

$$P_e = \sum_{i=1}^{3} p_i^A p_i^B = 0.424$$

$$\kappa = \frac{P_0 - P_e}{1 - P_e} = 0.983$$

The task-level performance for the cancerous class was:

$$\text{Sensitivity (TPR)} = \frac{3342}{3342 + 27} = 99.2\%$$

$$\text{Specificity (TNR)} = \frac{752 + 1991}{752 + 1991 + 11 + 24} = 0.990$$

Also, a single-threshold approximation to estimate AUC value (*Machine Learning* **2001**, *45(2)*, 171-186), and the AUC was computed as the mean of sensitivity (TPR) and specificity (TNR):

$$AUC_{single} = \frac{TPR + TNR}{2} \approx 0.990$$

Dataset	Training Dataset			Test Dataset		
	Tiles	Sections	Patients	Tiles	Sections	Patients
Healthy	440	32	11	109	6	3
Cancerous	2211	30	10	180	6	3

Table S10. Composition and partitioning of the revised NOS histopathology dataset, showing the number of tiles, tissue sections, and patients used for training and testing.

Model	Accuracy (%)	Precision (%)	Recall (%)	F1-score(%)	Cohen's Kappa
DenseNet121	95.5	95.6	97.2	96.4	0.904
ResNet50	89.6	90.1	96.1	93.0	0.728

Table S11. Performance of CNN models on the test dataset.

Cross-Val	Training Dataset			Test Dataset		
	Tiles	Sections	Patients	Tiles	Sections	Patients
Healthy	479	31	10	70	7	4
Cancerous	2211	30	10	180	6	3

Table S12. Composition and partitioning of another NOS histopathology dataset for cross validation, showing the number of tiles, tissue sections, and patients used for training and testing.

Model	Accuracy	Precision	Recall	F1-Score	Cohen's κ
Original	0.9550	0.9563	0.9722	0.9642	0.9037
Cross-Val	0.9100	0.8850	0.9833	0.9316	0.8013

Table S13. Quantitative comparison of model performance between the revised and cross-validation patient-level splits.
